# Investigating the impact of sex and reproductive aging on latent signatures of modifiable dementia risk factors

**DOI:** 10.1162/IMAG.a.967

**Published:** 2025-11-06

**Authors:** Alice Mukora, Manuela Costantino, Olivier Parent, Gabriel. A. Devenyi, M. Mallar Chakravarty

**Affiliations:** McGill University—Integrated Program in Neuroscience, Montreal, QC, Canada; Cerebral Imaging Centre—Douglas Mental Health University Institute, Montreal, QC, Canada; University of Chicago—Committee on Genetics, Genomics and Systems Biology, Chicago, IL, United States; Department of Psychiatry, McGill University, Montreal, QC, Canada

**Keywords:** sex differences, aging, structural MRI, menopause, partial least squares

## Abstract

Treating modifiable risk factors for dementia may serve to prevent maladaptive brain aging. These include, and are not limited to, obesity, hypertension, and other metabolic disorders. Nearly two-thirds of affected individuals are female, and emerging research has pointed to potential sex-specific factors, such as reproductive aging, as potent modifiers of dementia risk. Here, we leverage neuroimaging to characterize sex differences in the neural signatures of modifiable dementia risk factors, examining their relationship with brain anatomy, and integrate female-specific factors related to menopause history in a sex-specific analysis. Using cross-sectional data from the UK Biobank, we selected a cohort of 31,711 (age 44–82, 55.2% female/44.8% male) participants without diagnosed neurological conditions and collated behavioral data previously determined as modifiable risk factors. Using partial least squares analysis (PLS), we examined latent signatures that represent linear combinations that maximize covariance between patterns of brain (mean cortical thickness from 64 regions) and risk factor variables. To examine sex differences, we performed PLS analysis using the entire sample. We performed linear models to explore age-by-sex interactions with the PLS-derived brain scores and the risk factor pattern scores. To examine sex-specific relationships, we performed separate PLS analyses for the males and females and integrated menopause-related variables into the latter analysis. Our study found sex-dependent and menopause-dependent relationships between lifestyle risk factors and cortical thickness, highlighting stronger impacts of cardiometabolic factors on males and social and obesity-related factors on females in preserving brain health. The inclusion of menopause-related variables did not change the relationships with lifestyle risk factors, and strict age matching dampened the strength of the findings. Our findings suggest that lifestyles and the female-specific endocrine environment influence sex differences in cortical anatomy during brain aging.

## 1 Introduction

As the global population continues to age, public health concerns related to disease and maladaptive aging are on the rise (Government of Canada, 2022; Nichols et al., 2022). One such concern is dementia, a family of progressive neurodegenerative disorders that impair cognitive function. Despite extensive research, current treatments for dementia have not been effective in halting or slowing the progression of the disease, and no cure is currently available (Fessel, 2023). This has prompted investigation into modifiable risk factors that may serve as a promising arena to delay or prevent maladaptive neurodegeneration. A variety of risk factors, including obesity, hypertension, and smoking, have been found to account for 40% of global incidence (Livingston et al., 2020) and may serve as effective targets for risk reduction (Grodstein et al., 2023). While age is the primary risk factor for dementia, women are disproportionately affected by dementia, accounting for nearly two-thirds of affected individuals and having double the lifetime risk compared with men (Alzheimer’s Association, 2023). Historically, this higher incidence has been attributed to women’s longer life expectancy relative to men. However, emerging research suggests sex-dependent associations of risk factors with dementia incidence, cognitive decline, and neurodegeneration (Conde et al., 2021; Huo et al., 2022; Rocca et al., 2014). Profiles of dementia risk may be different between males and females due to sex differences in the prevalence of behaviors and environmental factors (Xiong et al., 2025), such as higher educational attainment and alcohol consumption (White, 2020; Wilsnack & Wilsnack, 2013) in older men. Sex also modulates the interactions with risk factors that have been associated with accelerated brain aging in women, such as waist-to-hip ratio (WHR) and body mass index (BMI) (Subramaniapillai et al., 2022, 2024); in contrast, lower educational attainment and former alcohol consumption have stronger associations with dementia incidence in men (Gong et al., 2023).

In addition to the above-mentioned sex–lifestyle interactions, women also possess unique risk factors, including those tied to reproductive history and reproductive aging (Azad et al., 2007; Than et al., 2022; Xiong et al., 2025). The menopause transition (MT), a neuroendocrine aging process specific to females, is understudied in this context. The MT has been linked to alterations in brain structure, particularly in areas involved in higher cognitive processes, although some studies have suggested that these changes stabilize post-menopause (Mosconi et al., 2021; Ramli et al., 2023). Early timing of the menopause onset, either through spontaneous or surgical means, has been associated with increased risk for cognitive decline (Liao et al., 2023). Other endogenous or exogenous alterations to sex-hormone concentrations, such as pregnancy and the use of menopausal hormone therapy (MHT), can also negatively impact cognitive function and increase dementia risk in later life (Barth et al., 2024; Barth & de Lange, 2020; Crestol et al., 2024). In attempts to account for the inherent age differences between menopausal groups, studies from our group have utilized age-matched menopause groups and have found attenuated or undetectable differences in neuroanatomy and cognition between menopausal groups (Costantino et al., 2023; Wezel et al., 2024). These findings suggested that while menopause-specific changes may play a role in dementia risk, careful consideration should be taken in isolating the effect of the menopausal transition from the effect of aging.

In this paper, we utilize a large sample from the UK Biobank to investigate the associations between modifiable dementia risk factors and brain anatomy in mid-to-late life, both at a population level and with a sex-specific focus. Furthermore, in females, we investigate how these associations vary according to menopause status and type. We leveraged neuroimaging-derived cortical thickness measures to non-invasively quantify brain morphology and investigated associations with a wide array of dementia risk factors, aiming to identify distinct anatomical patterns linked to risk factor profiles across chronological aging and changes in the reproductive system. Although our primary focus is on modifiable dementia risk factors, we also conducted secondary analyses incorporating polygenic risk for Alzheimer’s disease to examine whether our findings were robust to genetic susceptibility.

## 2 Materials and Methods

### 2.1 Sample

Cross-sectional data were obtained from the UK Biobank, a large-scale open-access multimodal biomedical database (Application #45551). Full details of recruitment and other key UK Biobank procedures have been published and are available on www.ukbiobank.ac.uk. All participants provided written informed consent. An extensive magnetic resonance imaging (MRI) protocol was acquired for ~40,000 participants between 2014 and 2020. Extensive data related to health and lifestyle were also acquired. We selected a cohort of 31,711 participants (age 44–82, 55.2% female/44.8% male) (Table 1) with available imaging data and passed the quality control metrics outlined below. In total, 14,428 female participants had complete data related to the reproductive aging analyses.

**Table 1.** Basic demographic information for the participants in each analysis.

	N	Age (years)*
**Sex Differences**		
Females	17,594	63.08 (7.03)
Males	14,661	63.88 (7.58)
**Sex Specific**		
Females	14,428	63.28 (6.72)
Males	14,208	63.82 (7.57)
**Menopause**		
Premenopausal	222	53.42 (2.39)
Natural Menopause	445	53.78 (2.67)
Surgical Menopause	223	54.11 (2.95)
Age-Matched Males	890	53.00 (2.64)

* Mean (Standard Deviation).

### 2.2 Image processing

UK Biobank used a standard 3T Siemens Skyra with a standard Siemens 32-channel RF receive head coil across three dedicated imaging centers in the United Kingdom (Miller et al., 2016). We performed manual quality control for motion on the T1-weighted (MPRAGE, 1 mm^3^) scans before image processing to avoid biases in morphometric measurements in subsets of the population, a process developed by our group (https://github.com/CoBrALab/documentation/wiki/Motion-Quality-Control-(QC)-Manual) (Bedford et al., 2020). The passed scans were input into CIVET 2.1.1, a cortical thickness estimation pipeline developed for human magnetic resonance (MR) images (Ad-Dab’bagh et al., 2006). Each scan was registered from native to stereotaxic space, using the MNI ICBM152 model as the registration target. Next, CIVET performed tissue classification into white matter (WM), gray matter (GM), and cerebrospinal fluid (CSF) based on the T1w image. WM and pial surfaces, composed of 40,962 vertices, were extracted for each hemisphere. Cortical thickness was calculated in millimeters as the distance between the pial and WM surfaces in the native space of the original image. Regional average cortical thickness values were then derived using the Desikan–Killiany–Tourville (DKT) atlas (Desikan et al., 2006; Klein & Tourville, 2012) applied to imaging data smoothed with a 30 mm full width at half maximum (*fwhm*) kernel. Finally, all cortical thickness outputs underwent a manual quality control step to ensure accurate tissue classification (https://github.com/CoBrALab/documentation/wiki/CIVET-Quality-Control-Guidelines).

As the project aims to explore healthy aging, participants with confounding neurological, psychiatric, and substance-related diagnoses that are known to impact neuroanatomy (n = 852) were excluded from analyses. Exposures were derived from self-report of illness or disability (UK Biobank Data Field 20002), and specific codes used for exclusion are detailed in Supplementary Table S1.

### 2.3 Lifestyle variables

At each visit to the assessment center, participants provided information through questionnaires about alcohol, total household income, smoking, hearing difficulty, physical activity, sleep, social support, and social isolation. We reviewed the literature to identify available variables that aligned with the modifiable dementia risk factors outlined in the 2020 Lancet Commission report, covering cardiometabolic risk (hypertension, diabetes, smoking, obesity, high alcohol consumption, and physical inactivity), behavioral factors (depression, low educational attainment, low social contact), and other conditions (hearing impairment) (Livingston et al., 2020). Additionally, we conducted a manual keyword search to identify further relevant variables of interest that have been related to dementia risk, including sleep disturbances (Benito-León et al., 2009; Lutsey et al., 2018; Sindi et al., 2018) and markers of socioeconomic status (Hunt et al., 2020; Kivimäki et al., 2020; Livingston et al., 2020). We then assessed the relevance and alignment of each variable with the domains and life course stages associated with the behaviors implicated in dementia risk. For instance, excessive alcohol consumption is considered a midlife risk factor (Anttila et al., 2004; Strandberg et al., 2018), so we chose to assess current alcohol consumption status and frequency, while discarding information about previous alcohol consumption and abstention. Preference was given to answers provided during the imaging visit.

Variables were excluded if more than 10% of the data were missing or if visual inspection revealed insufficient variance, defined as heavily skewed distributions concentrated in a single response category. This qualitative approach was used to ensure that all included variables contributed meaningful variability to the multivariate models. Additionally, any participant missing data for more than 10 demographic variables (n = 218) was excluded from analyses. Where appropriate, missing data were backfilled from visits preceding the imaging visit (n = 12,756). Across the retained dataset, 2% of values were missing, with variable-level missingness ranging from less than 1% to 11%. The remaining dataset showed no systematic patterns of missingness, based on visual inspection of matrix and bar plots. Missing data were then imputed using the missForest package (version 1.5) in R 4.2.2, with default parameters and parallelization across forests (parallelize = “forests”).

The categories used in our analyses were as reported in the UK Biobank questionnaire, unless otherwise noted. Categorical variables were dummy coded by assigning each level a discrete integer (e.g., 0, 1, 2, 3 for a four-level variable). For example, household income was coded on a scale from 1 to 5, with higher values indicating higher income brackets. Sleep chronotype was coded as a dichotomous variable, with 1 indicating “morning” types and 2 indicating “evening” types. Supplementary Table S2 contains the original names and codes of the fields from which all variables were derived. Supplementary Table S3 to S5 detail the demographics for all samples used in analyses.

#### 2.3.1 Genetic variables

Polygenic risk scores (PRS) for Alzheimer’s disease were included as a genetic risk measure, based on the Standard PRS set described by Thompson et al. (2022). These scores were derived using external GWAS data and made available by Genomics PLC under UK Biobank project 9659. PRS values were merged with the main dataset by participant ID and incorporated as continuous variables in statistical analyses. Supplementary Figure S7 shows the distributions of PRS scores across analysis groups. Further details on PRS derivation and validation are provided in Thompson et al. (2022).

### 2.4 Female-specific variables

Based on self-report data, female participants were divided into premenopausal women (PRE), women who underwent a bilateral oophorectomy (with or without a hysterectomy) prior to menopause (SURG), and women who underwent menopause without surgical interference (POST). Menopause status was assessed by self-report at each imaging visit, indicated as an answer of “Yes” or “No” to “Have you had your menopause (periods stopped)?” Surgical menopausal status was determined by self-report of bilateral oophorectomy (with or without a hysterectomy) prior to menopause (SURG) and women who underwent menopause without surgical interference (POST). Any participant who reported an age at menopause at the imaging time point or any previous visit to an assessment center, or was over 70 years old, was coded as postmenopausal. Surgical menopausal status was determined by self-report of bilateral oophorectomy and reported age at bilateral oophorectomy being at or prior to reported age at menopause. Any participant with uncertain menopause status or hysterectomy without a bilateral oophorectomy was excluded from any female-specific analysis. Binary (yes/no) variables were generated for analysis, encoding menopause status (premenopausal or postmenopausal), surgical menopause, or natural menopause.

### 2.5 Statistical analysis

#### 2.5.1 Group differences

We tested the interaction of female sex and age in cortical thickness measures in the whole sample (Eq. 1) using vertex-wise linear models in R/4.2.2:

VertexCortical Thickness ~ Age + Sex + Age:Sex (1)

All continuous variables were z-scored to obtain standardized beta coefficients. Results were corrected with the False Discovery Rate (FDR) (Benjamini & Hochberg, 1995) and are presented with a 5% FDR correction.

To investigate group-level differences in neuroanatomy within the female sample, we employed a vertex-wise analysis of variance model to test the interaction between age and menopause status (Eq. 2).

$$\begin{array}{l} Vertex\;Cortical\;Thickness\;\sim \;Age\;+\;Menopause\;Status\\ \;\;\;+\;Age:Menopause \end{array}$$ (2)

Results were FDR corrected and presented with a 20% FDR threshold owing to the limited findings in this analysis.

#### 2.5.2 Partial least squares analysis (PLS)

**Fig. 1.**
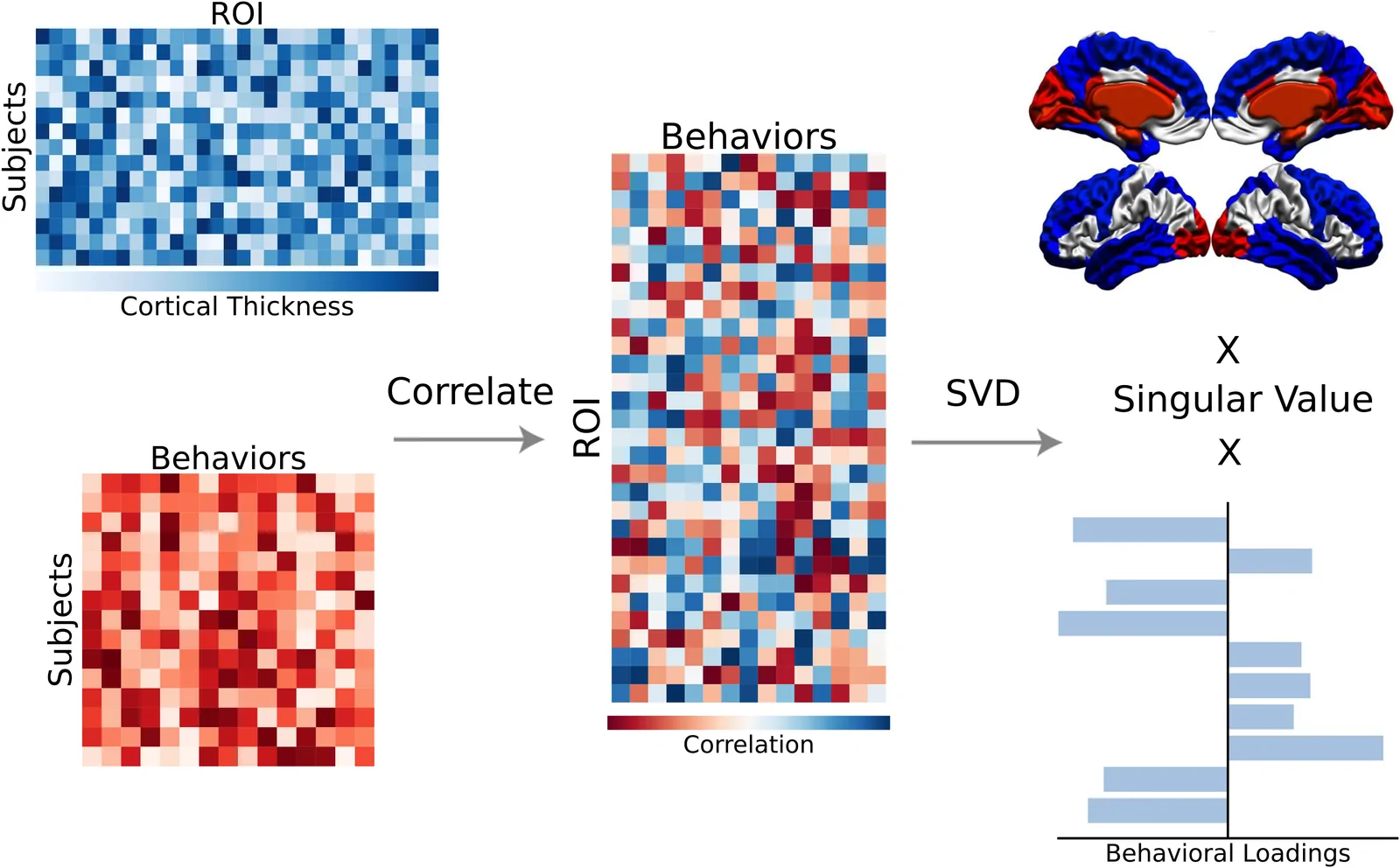
Workflow for partial least squares (PLS) analysis. PLS was used to identify patterns of covariance between regional cortical thickness measures and expression of lifestyle risk factor behavior for each participant in the analysis.

In order to assess the multivariate relationships between cortical thickness measures and risk factor behaviors, we performed partial least squares (PLS) analysis, a technique that extracts patterns of covariance between two sets of variables (McIntosh & Lobaugh, 2004; McIntosh & Mišić, 2013) (Fig. 1). PLS is particularly well suited to neuroimaging applications, as it models many-to-many relationships between neural and behavioral variables, offering a multivariate extension of mass univariate approaches (Danyluik et al., 2025; McIntosh et al., 1996) and facilitating robust brain–behavior mapping in large, high-dimensional datasets in large-scale population datasets such as the UK Biobank (Genon et al., 2022; Mihalik et al., 2022). The variables used were the brain matrix, which contained regional cortical thickness at each DKT atlas ROI, and the risk factor data matrix across all analysis-specific groupings of participants. These two matrices were standardized (z-scored) across each column, such that each variable—including continuous and dummy-coded categorical variables—had a mean of 0 and a standard deviation of 1, and then the covariance matrix was computed. This matrix was subjected to singular value decomposition, which yielded a set of latent variables (LVs)—linear combinations of the variables in the brain and behavioral variables that maximally covary with one another (Zeighami et al., 2019). For each LV, each participant has a calculated brain and behavior score, defined as the singular value obtained from the decomposition of the respective matrix, which represents the extent to which the participant expresses the cortical thickness and risk factor patterns of the LV. All analyses were performed using Pyls, a Python implementation of PLS (https://github.com/rmarkello/pyls).

The statistical significance of each LV was assessed through permutation testing (5,000 permutations), where the rows of the brain matrix are randomly shuffled and the LV is recomputed (Krishnan et al., 2011). The reliability of the contribution of each brain and behavior variable was calculated through bootstrap resampling (5,000 bootstraps). This process takes samples, with replacement, from the participants and reassesses the contribution of each variable to the LV to ensure that the pattern is not solely dependent on which participants are included in the sample. This reliability is measured as a bootstrap ratio (BSR), which is calculated by multiplying the weight of the variable in the LV by the singular value of that LV and dividing by the standard error (McIntosh & Lobaugh, 2004). A BSR of 1.95 was used, as this is analogous to a p-value of 0.05.

Additionally, we assessed the split-half stability of the LVs using a split-half resampling method, in which, for each PLS analysis, we split the sample into two halves, matching for age, sex, and menopause status where appropriate, and ran PLS on each half. For each resulting PLS model, we calculated the Pearson correlation between the cortical thickness and risk factor loadings separately (e.g., correlation between risk factor loadings for the first half and the second half). We took the absolute value of each correlation coefficient to account for arbitrary sign flips and repeated this process for 200 iterations. Finally, we used a metric proposed by McIntosh (2022) and Nakua et al. (2024) to determine whether the resulting Pearson correlation coefficient distributions were significant, suggesting consistent relationships regardless of the characteristics of participants. For each distribution, we performed a *Z*-test (i.e., mean divided by standard deviation of distribution) and a *Z*-test magnitude greater than 1.96, which is associated with a p value <0.05, indicating that the distribution significantly differed from 0. Results are provided in Supplementary Figure S6.

##### 2.5.2.1 Sex differences analysis

We performed PLS using the entire sample to elucidate common signatures of relationships between vertex-wise cortical thickness and lifestyle factors across sexes. All regression analyses were performed using R/4.2.2. Age and sex were not included in our input matrices, and the impact was examined post hoc using linear regression models to test the interaction of sex and age on PLS-derived brain and behavior scores (Eq. 3).

PLS−derived Score ~ Age+Sex+Age:Sex. (3)

##### 2.5.2.2 Sex-specific analysis and the impact of menopause status

To explore sex-specific relationships, we performed sex-specific PLS, separating males and females and using identical lifestyle risk factors. This approach aimed to highlight any unique patterns within each sex that might not be observable in a combined sample. The entire male cohort was analyzed, but only female participants with a reported menopause status were included (n = 14,428) (Costantino et al, 2023). To examine the relationship of these sex-specific patterns with the aging process, we ran linear models using PLS-derived scores as the dependent variable and age as the independent variable (Eq. 4).

PLS−derived Score ~ Age. (4)

In females, to determine the effects of menopause status, we ran linear models to determine whether there were interactions between menopause status and age on the PLS-derived scores (Eq. 5).

$$\begin{array}{l} PLS-derived\;Score\;\sim \;Age+Menopause\;Status\\ \;\;\;+Age:Menopause\;Status. \end{array}$$ (5)

Additionally, to account for sex-specific factors, we repeated the female-specific PLS with the addition of binary menopause, surgical menopause, and natural menopause variables described above.

##### 2.5.2.3 Sex-specific, age-matched analysis

As age is the most significant risk factor for the development and progression of dementia, as well as for the onset of menopause, it is challenging to disentangle the effects of chronological aging from those of reproductive aging. Both processes contribute to neuroanatomical and cognitive changes, but they may do so through distinct mechanisms. For a more focused exploration of reproductive aging, rather than chronological aging, the menopause groups were age matched using the nearest neighbor’s algorithm with the MatchIt package in R 4.1.2. An additional group of males was age matched to the final female cohort. We performed PLS on the female group alone and with the male age-matched group included. In both analyses, an ANOVA was conducted to determine group differences in brain and risk factor pattern scores in any significant LVs found.

##### 2.5.2.4 Sex-specific, age-matched analysis

As age is the most significant risk factor for the development and progression of dementia, as well as for the onset of menopause, it is challenging to disentangle the effects of chronological aging from those of reproductive aging. Both processes contribute to neuroanatomical and cognitive changes, but they may do so through distinct mechanisms. For a more focused exploration of reproductive aging, rather than chronological aging, the menopause groups were age matched using the nearest neighbor’s algorithm with the MatchIt package in R 4.1.2. An additional group of males was age matched to the final female cohort. We performed PLS on the female group alone and with the male age-matched group included. In both analyses, an ANOVA was conducted to determine group differences in brain and risk factor pattern scores in any significant LVs found.

##### 2.5.2.5 Influence of genetic risk

In supplementary analyses, we included PRS for Alzheimer’s disease as an additional predictor. We tested for main effects and interactions between PRS, age, and sex on PLS-derived brain and behavior scores for the whole sample analysis (Eq. 6) and between PRS and age in the sex-specific analysis (Eq. 7).

PLS−derived Score ~ Age+Sex+PRS + Age:Sex:PRS. (6)

PLS−derived Score ~ Age+PRS + Age:PRS. (7)

## 3 Results

### 3.1 Group differences

**Fig. 2.**
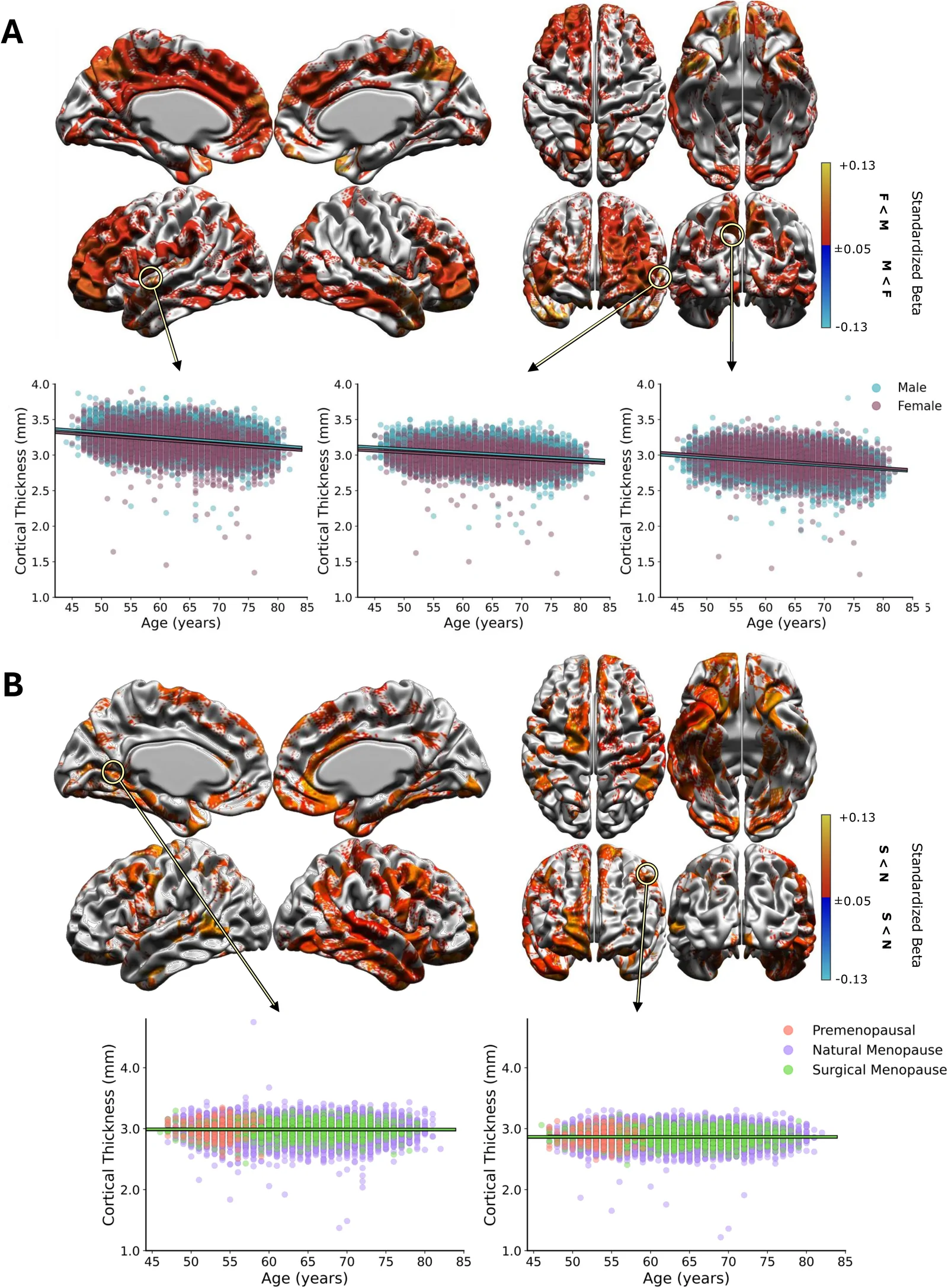
Results of linear regression analysis for group differences. Brain heatmaps showing significant age by female sex interaction, (A) and age by menopause status (B), colors indicate standardized beta coefficients. Plots of peak vertices are shown in the second row, stratified by sex and menopause status, respectively. Female sex*age at 5% FDR and menopause status*age at 20%FDR (**F:** Female; **M:** Males; **N:** Natural menopause; **S:** Surgical menopause)

A significant interaction between age and sex was observed over widespread regions of the cortex (Fig. 2A), demonstrating age-related differences in cortical thickness that varied significantly by sex (p(FDR) < 0.05). More pronounced age-related cortical thinning was found in females than in males in the frontal and temporal lobes, with the effect sizes (standardized beta coefficients) ranging from 0.023 to 0.116.

For the female sample, a significant interaction (FDR-corrected p < 0.05) between age and menopause status was only observed between the surgical menopause and the natural menopause groups (Fig. 2B), demonstrating thinner cortex in the surgical menopause group with age, relative to the natural menopause group, with effect sizes ranging from 0.05 to 0.13. These differences were most widespread in the right hemisphere, covering the temporal, inferior parietal, and middle frontal areas. In the left hemisphere, effects were more sparse but more pronounced in the caudal middle frontal area. Because the menopause-stratified analysis was based on a smaller age-matched subsample, results are presented at a 20% FDR threshold, with additional thresholds and unthresholded maps provided in Supplementary Figure S10.

### 3.2 Partial least squares analysis

**Fig. 3.**
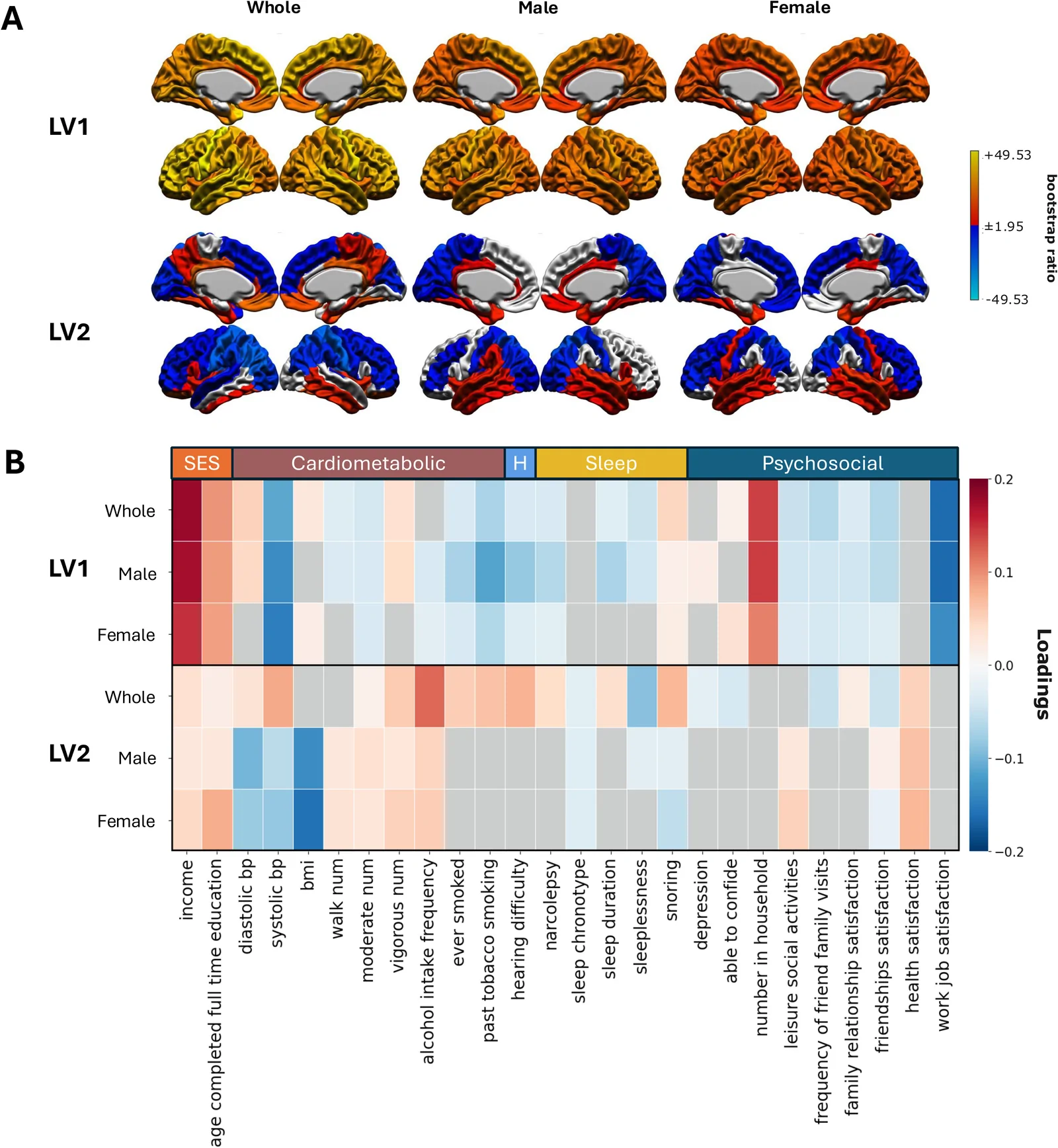
Results of whole sample, male-specific, and female-specific analyses. Morphometric (A) and risk factor (B) patterns for LV1 (top) and LV2 (bottom). Warm colors on morphometric maps indicate regions that vary positively with the LV, and cold colors indicate regions that vary negatively. These values are thresholded at ± 1.95 bootstrap ratio (95% confidence interval). The x-axis on the risk factor plot indicates individual risk factors, and the color indicates the direction of the association with LV. Warm colors indicate positive association and cooler colors indicate negative loadings. Gray boxes indicate non-significant factors, as assessed by bootstrapping. (H: Hearing Difficulty).

For all PLS analyses, detailed interpretation was limited to the first two statistically significant latent variables, or to those that together explained over 95% of the covariance in the analysis. This approach prioritizes the most stable and meaningful components of the brain–risk factor relationship, consistent with common practice in neuroimaging applications of PLS. Figure 3A and B displays the brain and behavioral patterns for the corresponding latent variables in the whole sample and sex-specific analyses that include only the lifestyle risk factors. Figure 3C and D displays the results for their respective linear regression models. In processing these analyses, we aimed to identify similarities and differences between the whole sample analysis and the sex-specific analyses, as well as between the sex-specific analyses themselves. There are multiple manifestations of differences that were observed: (1) factors that were significant in the whole sample analysis, but were not significant in either the male- or female-specific analyses; (2) factors that emerged only in the sex-specific analyses and not in whole sample analysis; and (3) factors that appeared exclusively in the analysis of one sex, but were absent in the other sex or the whole sample.

#### 3.2.1 Whole sample analysis

The PLS analysis yielded seven significant (p < 0.05) LVs (Supplementary Fig. S1), and the cortical thickness and risk factor loadings for the first five LVs were reproducible in split-half analysis (Supplementary Fig. S6). LV1 explained 91.71% (p < 0.001) of the covariance and was associated with significant contributions from factors related to preserved brain health, including higher socioeconomic status (e.g., increased educational attainment and higher income), more frequent vigorous exercise, and a history of no smoking. In contrast, maladaptive sleep-related factors (e.g., shorter sleep duration and snoring), as well as lower indicators of social interaction (e.g., fewer leisure and social activities) and subjective well-being (e.g., lower satisfaction in friendships, family relationships, and work), were also significant contributors. Other significant contributions included higher diastolic blood pressure, lower systolic blood pressure, higher BMI, lower frequency of walking and moderate exercise, fewer experiences of sleeplessness, and feeling able to confide in others. The covarying morphometry pattern involved positive associations with cortical thickness across the whole brain, strongest in the precentral gyrus, superior frontal gyrus, superior, and transverse temporal regions.

LV2 explained 4.36% (p < 0.001) of the covariance and demonstrated significant contributions more associated with greater dementia risk, including worsened cardiometabolic health (e.g., higher diastolic and systolic blood pressure), increased substance use (e.g., increased alcohol consumption and a history of smoking), and hearing difficulty. Significant sleep-related contributions were mixed, encompassing both improvements in sleep quality (e.g., longer sleep duration, earlier sleep chronotype, and reduced sleeplessness) and disturbances in sleep patterns (e.g., snoring and narcolepsy). Social contact and satisfaction contributions were also mixed, with greater satisfaction in health and family relationships, but feeling less able to confide in others, fewer visits from friends and family, and lower satisfaction with friendships. Contributions of higher socioeconomic status (e.g., increased educational attainment and higher income), exercise, and snoring were similar to those observed in LV1. Other significant contributions included an increased frequency of vigorous and moderate exercise, as well as an absence of depression. The covarying morphometric pattern included negative associations with cortical thickness across most of the frontal, parietal, and occipital regions, with the strongest associations in the postcentral gyrus regions of both hemispheres, as well as in the inferior parietal region of the left hemisphere and the superior parietal region of the right hemisphere. Both hemispheres had strong positive associations in the posterior cingulate, isthmus cingulate, and medial and lateral orbitofrontal areas.

**Fig. 4.**
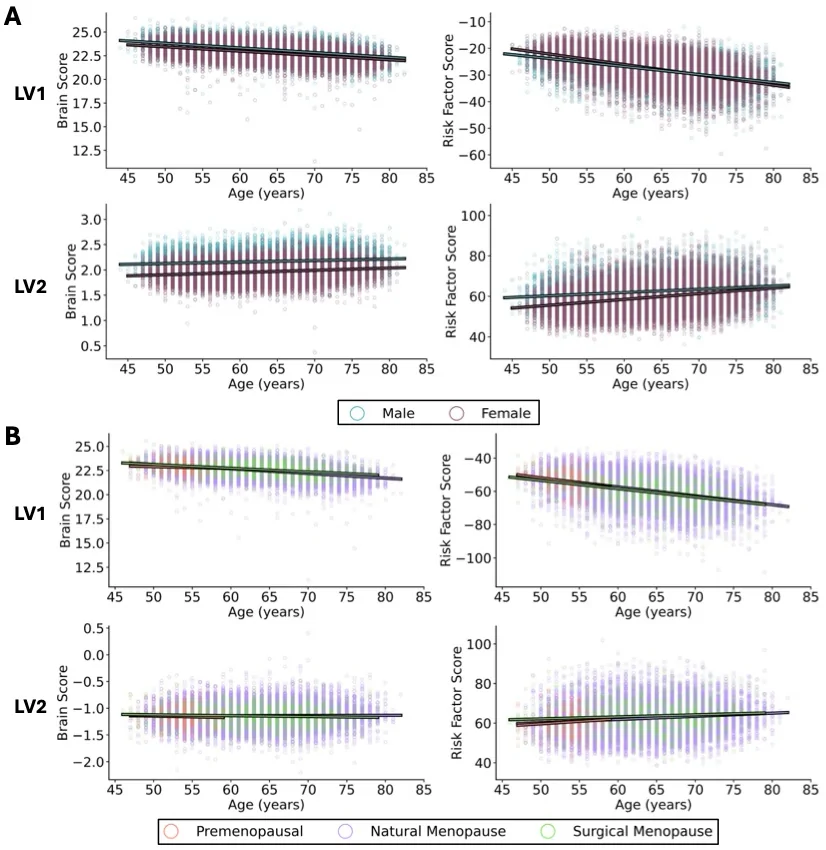
Interaction analyses of PLS-derived LV scores. Linear models used to test sex differences in the whole sample analysis (A) and menopause type differences in the female-specific analysis (B) for each LV-derived brain and risk factor scores for LV1 (top row) and LV2 (bottom row).

The regression analysis revealed significant (p < 0.05) main effects of age and sex and a significant interaction between them on brain and behavior scores in both LV1 and LV2 (Fig. 4A). For LV1, a negative main effect of age was observed for both brain (β = -0.424, p < 0.001) and behavior scores (β = -0.421, p < 0.001) on cortical thickness, suggesting these patterns are more strongly associated with younger individuals. Sex showed opposing effects on brain and behavior scores, with a negative main effect on brain scores (β = -0.392, p < 0.001), suggesting stronger expression of the LV1 morphometric pattern in males, and a positive main effect on behavior scores (β = 0.091, p < 0.001), suggesting a stronger expression of the LV2 pattern in females. The interaction of age and sex also differed by domain. For brain scores, the interaction was positive (β = 0.340, p = 0.0008), indicating that with increasing age, females exhibited a more pronounced decrease in brain scores. In contrast, the interaction was negative for behavior scores (β = -0.128, p < 0.001), indicating that males exhibited a rapid decrease in behavior scores for this pattern.

For LV2, a positive main effect of age was observed for brain scores (β = 0.104, p < 0.001) and behavior scores (β = 0.175, p < 0.001) on cortical thickness, suggesting these patterns are more strongly associated with older individuals. The opposite was observed for sex, with negative main effects observed for both brain scores (β = -0.965, p < 0.001) and behavior scores (β = -0.445, p < 0.001), suggesting stronger expression of both patterns in males. The interaction of age and sex was positive for both brain scores (β = 0.490, p < 0.001) and behavior scores (β = -0.130, p < 0.001), indicating that, with increasing age, females showed a more pronounced increase in brain scores and behavior scores for this pattern.

#### 3.2.2 Sex-specific analysis

##### 3.2.2.1 Male-specific analysis

In males, the PLS analysis yielded four significant (p < 0.05) LVs (Supplementary Fig. S2), and the cortical thickness and risk factor loadings for the first three LVs were reproducible in split-half analysis (Supplementary Fig. S6). LV1 accounted for 95.12% of the covariance (p < 0.05), with its significant risk factors and morphometric patterns closely resembling those identified in the whole sample analysis. Contributions from socioeconomic class, exercise, blood pressure, subjective well-being, and social enrichment remained consistent with the whole sample findings. However, the influence of snoring was reduced, while smoking history, hearing difficulty, and sleep disturbances showed stronger contributions. Depression and reduced alcohol intake frequency emerged as significant factors, whereas BMI and feeling able to confide were no longer significant. The associated morphometric pattern largely overlapped with that of the whole sample, showing positive associations with cortical thickness across the brain but with reduced loadings in the anterior cingulate regions.

LV2 accounted for 2.66% of the covariance (p < 0.05) and was linked to a risk factor profile markedly distinct from that of the whole sample analysis, showing a stronger association with preserved brain health. Significant contributions included greater educational attainment, higher income, better cardiometabolic health (e.g., lowered diastolic and systolic blood pressure, lower BMI, and more frequent exercise), better sleep (e.g., less frequent sleeplessness, no snoring), and greater social interaction and satisfaction (e.g., more leisure and social activities, greater satisfaction with friendships, and health). No significant associations were observed related to any smoking-related risk factors, though alcohol intake frequency was positively associated with this pattern. There is overlap with regions identified in the whole sample analysis, particularly in the middle frontal, parietal, and occipital regions, but many of the effects are dampened. Some regions exhibit a reversal in the direction of cortical thickness changes when analyzed specifically in males (e.g., cortical thickness increases in precuneus, temporal, and occipital regions), and other areas no longer have significant associations with the pattern (e.g., anterior cingulate, prefrontal gyrus, precentral gyrus, rostral middle frontal, and superior frontal regions).

The results of the regression analysis revealed significant negative effects of age on brain scores in LV1 (β = -0.440, p < 0.001) but not in LV2 (β = -0.0898, p = 0.284). However, age was a significant predictor of behavior scores in both LV1 (β = -0.4191, p < 0.001) and LV2 (β = -0.026, p = 0.00182), showing a decline with increasing age and stronger associations with younger individuals.

##### 3.2.2.2 Female-specific analysis

In females, the PLS analysis with only lifestyle risk variables yielded five significant (p < 0.05) LVs (Supplementary Fig. S3), and the cortical thickness and risk factor loadings for the first five LVs are reproducible (Supplementary Fig. S6). LV1 explained 91.89% (p < 0.05) of the covariance and demonstrated similar behavioral contributions as LV1 in the whole sample analysis. Contributions from socioeconomic class, smoking history, exercise, hearing difficulty, and social enrichment remained consistent with the whole sample findings, but associations with sleep disturbances (e.g., sleep duration and sleeplessness) were not observed. As seen in the male analysis, lowered alcohol intake frequency emerged as a significant contributor, and BMI and feeling able to confide are retained from the whole sample analysis, which did not occur in the male analysis. There was no significant contribution from diastolic blood pressure, as observed previously, but a lowered systolic blood pressure remained significant. Additionally, a significant contribution of less frequent moderate exercise was found; however, no significant contribution was observed for walking or vigorous exercise, which were also present in the whole sample and male analysis. The covarying morphometry pattern involved the cortical thickness increased across all regions, overlapping with the whole sample and male analysis, with dampening in cingulate areas.

LV2 explained 4.73% (p < 0.05) of the covariance and demonstrated a similar risk factor pattern to LV2 of the male analysis, though no association with sleeplessness was observed, and the contribution of friendship satisfaction became negative. Many of the significant risk factor contributions were stronger in females, except for lower diastolic blood pressure and increased moderate exercise. The covarying morphometric pattern was also similar to that of LV2 in the male analysis, with retained associations with decreased cortical thickness in superior parietal areas and widespread associations with increased cortical thickness in temporal regions. Associations with reduced cortical thickness in frontal regions were retained from the whole sample analysis, but associations with occipital, cingulate, paracentral, and supramarginal regions were not observed.

The results of the regression analysis found significant negative effects of age for brain scores in LV1 (β = -0.039, p = 0.008) and LV2 (β = -0.006, p = 0.008), as well as for behavior scores in LV1 (β = -0.406, p = 0.008) and LV2 (β = -0.136, p < 0.001), showing a decline with increasing age and stronger associations with younger individuals.

Our menopause status and age analysis (Fig. 4B) found a significant main effect of surgical menopause on behavior scores for LV1 and LV2. In LV1, surgical menopause (β = -0.11177, p = 0.000753) and the interaction of age and surgical menopause (β = -0.1448, p < 0.001) had negative associations with brain scores relative to the postmenopausal reference group. In LV2, the effects of surgical menopause (β = 0.0863, p = 0.00923) and a significant interaction of age and surgical menopause (β = 0.0716, p = 0.0267) were positive, indicating that surgical menopause, both as a main effect and in its interaction with age, played a significant role in predicting behavioral scores. In contrast, premenopausal status and its interaction with age did not significantly influence behavioral outcomes. No other significant effects were found for the other terms in the model, or for any terms in the brain model.

Repeated PLS analysis with the addition of the binary menopause status variables yielded five significant (p < 0.05) LVs (Supplementary Fig. S4) and the cortical thickness and risk factor loadings for the first three LVs were reproducible (Supplementary Fig. S6). LV1 and LV2 demonstrated identical morphometric and risk factor patterns as those in the previous analysis. LV1 explained 92.11% (p < 0.05) of the covariance and demonstrated additional significant positive contributions of surgical menopause, but negative contributions of being premenopausal or experiencing natural menopause. LV2 explained 4.47% (p < 0.05) of the covariance and showed no significant contributions of any of the menopause variables.

### 3.3 Age-matched PLS analysis

In the age- and menopause status-matched female cohort, the PLS analysis with the inclusion of age-matched males found one significant LV (Supplementary Fig. S5, left) accounting for 59.84% of the covariance. Additionally, the risk factor loadings of the significant LV were found to be reproducible from split-half analysis, but the cortical

thickness loadings were not (Supplementary Fig. S6). This LV demonstrated significant contributions from less frequent walking, having more people in the household, and greater work satisfaction. The covarying morphometry pattern involved the cortical thickness increases across all regions. We did not find any statistically significant differences in either risk factor pattern scores (F(3) = 0.202, p = 0.895) or brain scores (F(3) = 1.024, p = 0.381) between the groups.

Repeated analysis with the exclusion of the male cohort yielded two significant (p < 0.05) LVs (Supplementary Fig. S5, right), and the cortical thickness and risk factor loadings were found to be reproducible for LV2, but not for LV1 (Supplementary Fig. S6). LV1 explained 60.89% (p < 0.05) of the covariance and demonstrated significant contributions of lower diastolic blood pressure, earlier sleep chronotype, and shorter sleep duration. The covarying morphometry pattern involved the cortical thickness increases across all regions. Neither brain scores (F(2) = 0.544, p = 0.581) nor behavior scores (F(2) = 0.664, p = 0.515) were different between the menopausal groups.

LV2 explained 9.45% (p < 0.05) of the covariance and demonstrated significant contributions of depression and ability to confide. The covarying morphometric pattern consisted of a decrease in cortical thickness in frontal regions and an increase in cortical thickness in temporal regions. Neither brain scores (F(2) = 0.052, p = 0.95) nor behavior scores (F(2) = 0.497, p = 0.609) were different between the menopausal groups.

### 3.4 Assessing the influence of Alzheimer’s genetic risk

In the whole sample models, the inclusion of PRS revealed limited evidence for a moderating effect of genetic risk on the brain–behavior associations. While some terms involving PRS reached significance (e.g., a modest PRS main effect in the LV2 brain model, p = 0.0022), no consistent pattern was observed across models or subgroups. Sex-stratified models likewise showed few significant PRS-related interactions. These findings suggest that, although genetic risk for Alzheimer’s disease may have a modest relationship with individual brain or behavior scores, the primary PLS associations identified in our main analysis appear robust to the inclusion of this non-modifiable genetic factor. Age-related trajectories of brain and behavior scores across PRS quartiles and subgroups are visualized in Supplementary Figures S8 and S9. Full model results are presented in Supplementary Table S6.

## 4 Discussion

In this study, we aimed to investigate the impact of sex and menopause on the associations between known modifiable risk factors and cortical thickness in healthy aging in a large, well-powered cohort of middle-aged and older adults. Overall, we found effects of sex and surgical menopause on age-related decline across the frontal and temporal lobes. We computed regression models to determine the interaction between age and sex, as well as between age and menopause status, revealing regions with greater age-related cortical thinning in women than in men, and in women who underwent surgical menopause than those who experienced natural menopause, in areas associated with aging and cognition. We performed a series of PLS analyses to identify patterns of covariance between cortical thickness and dementia risk factor behaviors and assessed their relationships with the aging trajectory. We detected patterns that suggested sex-specific impacts of risk factors on brain anatomy, as well as a complex interaction between chronological aging and reproductive aging that was not fully disentangled by strict age matching. Given the established influence of genetic factors such as APOE on dementia risk, we examined whether the inclusion of polygenic risk scores for Alzheimer’s disease modified our findings. The results of these supplementary models were largely consistent with our main analyses, suggesting that the observed brain–behavior relationships are not strongly confounded by common genetic risk. However, the potential interplay between genetic and modifiable risk factors remains an important area for future work.

Our approach of a combined whole sample PLS and sex-specific PLS allowed us to unravel sex-specific relationships with healthy brain aging that may have been diluted or obscured in siloed analysis. For LV1 in all main analyses, widespread increased cortical thickness was significantly associated with risk factor profiles generally linked to the preservation of brain health in younger individuals, but alcohol intake frequency was only identified as a significant impact in the sex-specific analysis. Heavy alcohol consumption has been linked to the cortical thickness of the precentral gyrus, superior temporal gyrus, and the dorsolateral prefrontal cortex (Morris et al., 2019), regions strongly associated with LV1 for all analyses, but it is possible that greater variability in alcohol consumption or outcomes within one sex diluted the overall signal in the pooled analysis. Though men tend to drink more frequently than women (Wilsnack et al., 2009), studies have found more severe neuroanatomical effects of alcohol substance use disorders in women, even with similar intake patterns (Seo et al., 2019; Srivastava et al., 2010; Verplaetse et al., 2021; White, 2020). Other factors showed an impact on one sex but not the other, such as BMI, which contributed significantly to this pattern in women but not in men. Body fat has been shown to have potential neuroprotective effects in women as a primary source of estrogen (Simpson, 2003), and similar measures of BMI in men and women may be indicative of differing distributions of adipose tissue post-puberty (Chang et al., 2018; Fried et al., 2015; Karastergiou et al., 2012). One study has suggested that measures of visceral adipose tissue, rather than BMI, serve as a better anthropometric measure of obesity in women, as they confer a much stronger association with cardiometabolic and cardiovascular risk (Kammerlander et al., 2021). The inclusion of binary menopause status factors in the female analysis did not alter the observed risk factor patterns and revealed contributions indicative of younger individuals in the sample, as both the premenopausal and surgical menopause groups were younger than the postmenopausal group. Additionally, many of the risk factor contributions retained from the whole sample analysis were stronger in males, relative to females, in ways that sex-related differences in the strength of impacts may explain. Household size, which can be a reliable proxy measure of social isolation, contributed substantially to both patterns and has been associated with cortical thickness in the frontal pole, temporal cortex, and precuneus cortex (Cong et al., 2024), but men have been shown to both experience more social isolation (Umberson et al., 2022) and experience more mental deterioration in isolation than women (Dury et al., 2021).

In contrast, the opposing morphometric and risk factor patterns of LV2 differed between the whole sample analysis and the sex-specific analysis, particularly in their relationship with age. LV2 exhibited associations between poorer cardiometabolic health and reduced cortical thickness in widespread regions associated with dorsal attention and executive systems, but increased cortical thickness in regions linked to emotional and motivational systems, such as the anterior cingulate cortex and orbitofrontal cortex—a latent dimension also observed in another study (Nicolaisen-Sobesky et al., 2024). This pattern was associated with older adults but was expressed more rapidly with increasing age in women, possibly due to the greater vulnerability of women to the effects of cardiometabolic risk factors on cortical thickness (Kim et al., 2019; Zhernakova et al., 2022). However, LV2 in the sex-specific analyses showed patterns of better cardiometabolic health and increased cortical thickness in temporal regions for both sexes, but a reduction in parietal and occipital regions in women and frontal and parietal regions in men. In both sexes, the brain pattern showed no significant relationship with age; however, the risk factor patterns exhibited a significant yet subtle relationship with age in males and a similar interaction between age and menopause status (surgical vs. natural menopause) in females. These findings concur with evidence that cardiometabolic influences on neural health emerge more gradually in men. Additionally, women who undergo surgical menopause, which has been shown to result in more prevalent cardiometabolic disturbances (Farahmand et al., 2015), may exhibit greater vulnerability in these areas to accelerated aging due to hormonal insufficiency.

Importantly, we observed that some variables, such as systolic and diastolic blood pressure, showed different loading directions in the combined versus sex-stratified PLS analyses. For example, while these variables loaded positively on LV2 in the whole sample model, they exhibited negative loadings in both male and female subgroups. This divergence reflects the context-specific nature of multivariate decomposition: in PLS, loadings capture the contribution of each variable to the latent structure that maximizes covariance between predictors and outcomes. When data are stratified by sex, the underlying covariance patterns—and, therefore, the latent variables themselves—can change direction, revealing distinct associations that may be masked in the analysis of the whole sample. Rather than indicating inconsistency, this highlights the value of stratified modeling for uncovering sex-specific relationships between risk factors and brain–behavior profiles. We caution that direct comparisons of loading directions across models should be made with attention to these shifts in covariance structure.

Our results from the PLS with strict age matching replicated previous findings showing that, when isolating the impacts of menopause at midlife, we find no differences between the menopause groups or their male counterparts in either risk factor patterns or behavioral patterns, which aligns with similar analyses from our group (Costantino et al., 2023; Wezel et al., 2024). For the females, this aligns with the findings of the PLS analyses with menopause variables included, which did not change any of the observed risk factor or morphometric patterns found in their absence. For LV1, the menopause-related variable contributions were indicative of younger individuals in the sample, as both the premenopausal and surgical menopause groups were younger than the postmenopausal group, which aligns with stronger association of that LV with younger individuals. Moreover, none of the menopause-related variables had significant contributions to any of the remaining LVs in that analysis. However, we did find significantly faster age-related cortical thinning in women who underwent surgical menopause in the vertex-wise models of cortical thickness, which may indicate that higher-resolution neuroanatomical measures may be necessary to detect menopause-related differences.

Although our primary PLS results demonstrated strong stability across split-half resampling procedures, we acknowledge that reduced sample sizes in stratified analyses (e.g., sex-specific and age-matched subsamples) may have limited statistical power. As a result, the patterns observed in these smaller analyses should be interpreted with caution. Future studies in independent cohorts will be important for validating these exploratory findings and further clarifying the sex- and age-specific relationships between modifiable risk factors and brain structure.

Importantly, these findings underscore the importance of tailoring public health strategies to sex-specific patterns of dementia risk. The stronger associations between cardiometabolic risk factors and cortical thinning in males suggest that early detection and intervention for conditions such as hypertension and obesity may be particularly impactful in this group. In contrast, the greater influence of social and obesity-related factors in females points to the importance of incorporating psychosocial dimensions into brain health interventions targeted at women. Notably, the lack of additional explanatory power from menopause-related variables in the multivariate models suggests that such interventions could be broadly applicable across menopausal subgroups. Altogether, these results support the need for personalized, sex-aware preventive approaches that address modifiable risk factors to preserve cortical integrity and promote healthy cognitive aging.

### 4.1 Limitations

First, a limitation of this study is the reliance on cross-sectional data from the UK Biobank, which precludes direct inferences about the mechanistic or causal nature of the latent associations identified and limits the ability to make definitive claims about their relationship with aging. Second, it has been demonstrated that biases exist in self-report accuracy in biobank-scale research (Schoeler et al., 2023). A large majority of the risk factor data were collected in the form of questionnaires, and self-reported levels of factors such as physical activity can be both underreported or overreported due to recall bias in older adults (Falck et al., 2020) or responses aimed at socially desirable responses (Althubaiti, 2016). Additionally, this study was limited to the characterization of each lifestyle risk factor available from the lifestyle information collected by the UK Biobank. Although extensive, it was found that the approach limited the ability to capture each risk factor or its dimensions fully. For example, depression is considered a risk factor for dementia, and our analysis was limited to a third-party derived variable of a probable major depression as a binary input variable. This “diagnosis” category was defined using items related to the lifetime experience of depressive symptoms (Smith et al., 2013). Moreover, many other variables were captured as categorical variables that were dummy coded to discrete integers, which may impact the ability of the PLS analysis to meaningfully explore the covariance patterns of the risk factor variables as would be possible with more continuous variables. This process could be improved by the addition of a more comprehensive set of behavioral variables that capture a wider range of aspects of each behavior and potential impacts on cortical thickness. Additionally, repeated analysis with male and female cohorts better matched across behavioral variables would allow us to explore the results of the PLS analysis more accurately in the context of sex differences. Finally, while our analyses focused on structural MRI, future work could benefit from integrating both structural and functional modalities. Functional MRI may capture early network-level disruptions in brain connectivity that precede detectable anatomical changes, offering a complementary perspective on the neural mechanisms underlying dementia risk.

Finally, it is critical that the field begin to examine the impact of sex-specific and sex-relevant factors with studies that are designed to capture population-level variability. In our analyses, we integrated variables related to menopause that are provided by self-report while seeking to account for the impact of age. While the impact of menopause-related variables had a modest impact on our outcome measures, we chose to report our findings with a more lenient statistical threshold. Nonetheless, given the sensitivity and importance of the research question, namely the relationship between menopause and brain variation, we felt it was important not to discard potentially meaningful information that could provide some evidence for future studies on the topic.

## Supplementary Material

Supplementary Material

## Data Availability

The data supporting this study’s findings are available through the UK Biobank application procedure (https://www.ukbiobank.ac.uk/enable-your-research/register); scripts are available from the authors upon request.
