# Supplementary material for: Investigating the impact of sex and reproductive aging on latent signatures of modifiable dementia risk factors
Source: Imaging Neurosci (Camb). 2025 Nov 6;3:IMAG.a.967. doi: 10.1162/IMAG.a.967 (PMC12592373; doi:10.1162/IMAG.a.967)
Supplement: Supplementary Material [file IMAG.a.967_supp.pdf]

S. Supplementary Materials

S1. Supplementary Tables

Non-cancer Illness Coding	Meaning	N
1081	Stroke	230
1082	Transient ischaemic attack (tia)	160
1083	Subdural haemorrhage/haematoma	7
1086	Subarachnoid haemorrhage	7
1240	Neurological injury/trauma	4
1243	Psychological/psychiatric problem	1
1244	Infection of nervous system	0
1245	Brain abscess/intracranial abscess	2
1246	Encephalitis	12
1247	Meningitis	92
1258	Chronic/degenerative neurological problem	2
1259	Motor neurone disease	3
1261	Multiple sclerosis	79
1262	Parkinson's disease	44
1263	Dementia/Alzheimers/cognitive impairment	8
1264	Epilepsy	116
1266	Head injury	31
1408	Alcohol dependency	19
1409	Opioid dependency	0
1410	Other substance abuse/dependency	0
1434	Other neurological problem	17
1491	Brain haemorrhage	12
1583	Ischaemic stroke	10
1626	Fracture skull / head	31

Table S1: Non-cancer illness codes used to exclude participants due to neurological, psychiatric, and substance-related diagnoses.

Final Variable Name	Data Field
Age	21003
Sex	31
Alcohol drinker status	20117
Alcohol intake frequency	1558
Diastolic blood pressure	94, 4079
Systolic blood pressure	93, 4080
Diabetes	2443
Age completed full time education	845, 6138
Hearing difficulty	2247, 2257
Hypertension	2966, 6150
Depression	20123, 20124, 20125
Waist-to-hip ratio	48, 49
Duration of walks	874
Duration of vigorous activity	914
Number of days/week walked 10+ minutes	864
Number of days/week of moderate physical activity 10+ minutes	884
Number of days/week of vigorous physical activity 10+ minutes	904
Average total household income before tax	738
Sleep duration	1160
Morning-evening person (chronotype)	1180
Sleeplessness/insomnia	1200
Snoring	1210
Daytime dozing/sleeping (narcolepsy)	1220
Smoking status	20116
Past tobacco smoking	1249
Pack years of smoking	20161
Able to confide	2110
Frequency of friend-family visits	1031
Number in household	709
Leisure-social activities	6160
Work-job satisfaction	4537
Health satisfaction	4548
Family relationship satisfaction	4559
Friendships satisfaction	4570
Medication for cholesterol, blood pressure or diabetes	6177
Ever smoked	20160
Medication for cholesterol, blood pressure or diabetes	6177
Ever smoked	20160

Table S2: Variable field codes from the UK Biobank used to derive final analysis variables

N	Female<br>17,503	Male<br>14,208	p-value
**Age (years)**	63.1 (7.0)	63.8 (7.6)	**< 0.001**
**Income**			**< 0.001**
Less than 18,000	2414 (13.8%)	1319 (9.3%)	
18,000 to 30,999	5237 (29.9%)	3559 (25.0%)	
31,000 to 51,999	5443 (31.1%)	4428 (31.2%)	
52,000 to 100,000	3441 (19.7%)	3720 (26.2%)	
Greater than 100,000	968 (5.5%)	1182 (8.3%)	
**Age completed full time education (years)**	18.9 (2.5)	19.1 (2.6)	**< 0.001**
**Diastolic Blood Pressure (mmHg)**	77.0 (9.4)	80.6 (9.3)	**< 0.001**
**Systolic Blood Pressure (mmHg)**	135.2 (17.9)	141.2 (16.2)	**< 0.001**
**Body Mass Index (BMI)**	26.0 (4.6)	27.0 (3.8)	**< 0.001**
**Frequency of walking**	5.8 (1.7)	5.6 (1.8)	**< 0.001**
**Frequency of moderate exercise**	4.1 (2.2)	3.9 (2.2)	**< 0.001**
**Frequency of vigorous exercise**	1.8 (1.8)	2.2 (1.9)	**< 0.001**
**Alcohol Intake Frequency**			**< 0.001**
Never	1330 (7.6%)	736 (5.2%)	
Special occasions only	2351 (13.4%)	950 (6.7%)	
One to three times a month	2330 (13.3%)	1339 (9.4%)	
Once or twice a week	4765 (27.2%)	3679 (25.9%)	
Three or four times a week	4404 (25.2%)	4566 (32.1%)	
Daily or almost daily	2323 (13.3%)	2938 (20.7%)	
**Ever Smoked**			**< 0.001**
No	8838 (50.5%)	6207 (43.7%)	
Yes	8665 (49.5%)	8001 (56.3%)	
**Past Tobacco Smoking**			**< 0.001**
I have never smoked	8791 (50.2%)	6185 (43.5%)	
Just tried once or twice	2806 (16.0%)	2333 (16.4%)	
Smoked occasionally	2327 (13.3%)	1724 (12.1%)	
Smoked on most or all days	3579 (20.4%)	3966 (27.9%)	
**Hearing Difficulty**			**< 0.001**
No	10694 (61.1%)	7079 (49.8%)	
Yes	6809 (38.9%)	7129 (50.2%)	
**Narcolepsy**			**< 0.001**
Never/rarely	13966 (79.8%)	10576 (74.4%)	
Sometimes	3187 (18.2%)	3263 (23.0%)	
Often	350 (2.0%)	369 (2.6%)	
All of the time	0 (0%)	0 (0%)	
**Sleep Chronotype**			0.226
Morning person	11748 (67.1%)	9444 (66.5%)	
Evening person	5755 (32.9%)	4764 (33.5%)	
**Sleep Duration**	7.1 (1.0)	7.2 (1.0)	
**Sleeplesness**			
Never/rarely	2765 (15.8%)	4076 (28.7%)	
Sometimes	8481 (48.5%)	6239 (43.9%)	
Usually	6257 (35.7%)	3893 (27.4%)	
**Snoring**			**< 0.001**
No	12777 (73.0%)	7999 (56.3%)	
Yes	4726 (27.0%)	6209 (43.7%)	
**Depression**			**< 0.001**

None	16135 (92.2%)	13437 (94.6%)	
Single episode of probably major depression	351 (2.0%)	196 (1.4%)	
Probable recurrent major depression (moderate)	716 (4.1%)	352 (2.5%)	
Probable recurrent major depression (severe)	301 (1.7%)	223 (1.6%)	
**Able to Confide**			0.530
Never or almost never	1655 (9.5%)	2450 (17.2%)	
Once every few months	1116 (6.4%)	945 (6.7%)	
About once a month	1146 (6.5%)	779 (5.5%)	
About once a week	2325 (13.3%)	1414 (10.0%)	
2-4 times a week	2149 (12.3%)	1183 (8.3%)	
Almost daily	9112 (52.1%)	7437 (52.3%)	
**Number in Household**	2.1 (1.0)	2.2 (1.0)	0.127
**Leisure/Social Activities**	1.3 (0.9)	1.1 (0.9)	0.407
**Frequency of Friend/Family Visits**			0.636
No friends/family outside household	16 (0.1%)	17 (0.1%)	
Never or almost never	132 (0.8%)	250 (1.8%)	
Once every few months	846 (4.8%)	1099 (7.7%)	
About once a month	1897 (10.8%)	2198 (15.5%)	
About once a week	5526 (31.6%)	5110 (36.0%)	
2-4 times a week	6494 (37.1%)	4367 (30.7%)	
Almost daily	2592 (14.8%)	1167 (8.2%)	
**Friendships Satisfaction**			0.759
Extremely unhappy	9 (0.1%)	25 (0.2%)	
Very unhappy	42 (0.2%)	48 (0.3%)	
Moderately unhappy	329 (1.9%)	452 (3.2%)	
Moderately happy	4627 (26.4%)	4879 (34.3%)	
Very happy	9729 (55.6%)	7306 (51.4%)	
Extremely happy	2767 (15.8%)	1498 (10.5%)	
**Health Satisfaction**			0.467
Extremely unhappy	88 (0.5%)	34 (0.2%)	
Very unhappy	220 (1.3%)	159 (1.1%)	
Moderately unhappy	1183 (6.8%)	828 (5.8%)	
Moderately happy	7744 (44.2%)	6327 (44.5%)	
Very happy	7140 (40.8%)	5908 (41.6%)	
Extremely happy	1128 (6.4%)	952 (6.7%)	
**Work/Job Satisfaction**			< **.001**
Extremely unhappy	777 (4.4%)	906 (6.4%)	
Very unhappy	3470 (19.8%)	3516 (24.7%)	
Moderately unhappy	3564 (20.4%)	3308 (23.3%)	
Moderately happy	1153 (6.6%)	572 (4.0%)	
Very happy	803 (4.6%)	139 (1.0%)	
Extremely happy	222 (1.3%)	69 (0.5%)	
I am not employed	7514 (42.9%)	5698 (40.1%)	

Table S3: Sample demographics of the whole sample, separated by sex

				p-value	
	**Premenopausal**	**Natural Menopause**	**Surgical Menopause**	**Pre vs Natural**	**Pre vs Surgical**
**N**	222	13,227	979		
**Age (years)**	53.4 (2.4)	63.4 (6.6)	63.7 (6.9)	**< 0.001**	**< 0.001**
**Income**				**< 0.001**	**< 0.001**
Less than 18,000	14 (6.3%)	1810 (13.7%)	127 (13.0%)		
18,000 to 30,999	28 (12.6%)	4021 (30.4%)	281 (28.7%)		
31,000 to 51,999	80 (36.0%)	4116 (31.1%)	364 (37.2%)		
52,000 to 100,000	72 (32.4%)	2584 (19.5%)	161 (16.4%)		
Greater than 100,000	28 (12.6%)	696 (5.3%)	46 (4.7%)		
**Age completed full time education (years)**	19.3 (2.0)	18.9 (2.5)	18.4 (2.6)	**0.020**	**< 0.001**
**Diastolic Blood Pressure (mmHg)**	76.2 (8.3)	76.9 (9.4)	77.5 (9.5)	0.288	0.071
**Systolic Blood Pressure (mmHg)**	126.0 (14.7)	135.2 (17.9)	136.5 (18.1)	**< 0.001**	**< 0.001**
**Body Mass Index (BMI)**	26.2 (4.6)	25.9 (4.5)	26.5 (4.4)	0.305	0.116
**Frequency of walking**	5.6 (1.9)	5.8 (1.6)	5.7 (1.8)	0.249	0.832
**Frequency of moderate exercise**	3.6 (2.2)	4.1 (2.2)	4.0 (2.2)	**< 0.001**	**0.013**
**Frequency of vigorous exercise**	1.9 (1.9)	1.9 (1.8)	1.7 (1.8)	0.650	0.183
**Alcohol Intake Frequency**				0.080	0.251
Never	10 (4.5%)	1002 (7.6%)	86 (8.8%)		
Special occasions only	30 (13.5%)	1698 (12.8%)	156 (15.9%)		
One to three times a month	36 (16.2%)	1739 (13.1%)	140 (14.3%)		
Once or twice a week	72 (32.4%)	3586 (27.1%)	274 (28.0%)		
Three or four times a week	44 (19.8%)	3434 (26.0%)	198 (20.2%)		
Daily or almost daily	30 (13.5%)	1768 (13.4%)	125 (12.8%)		
**Ever Smoked**				0.061	0.283
No	126 (56.8%)	6638 (50.2%)	514 (52.5%)		
Yes	96 (43.2%)	6589 (49.8%)	465 (47.5%)		
**Past Tobacco Smoking**				**0.036**	0.056
I have never smoked	126 (56.8%)	6607 (50.0%)	514 (52.5%)		
Just tried once or twice	40 (18.0%)	2141 (16.2%)	135 (13.8%)		
Smoked occasionally	27 (12.2%)	1781 (13.5%)	138 (14.1%)		
Smoked on most or all days	29 (13.1%)	2698 (20.4%)	192 (19.6%)		
**Hearing Difficulty**				**< 0.001**	**< 0.001**
No	163 (73.4%)	8080 (61.1%)	586 (59.9%)		
Yes	59 (26.6%)	5147 (38.9%)	393 (40.1%)		
**Narcolepsy**				0.535	0.811
Never/rarely	183 (82.4%)	10550 (79.8%)	792 (80.9%)		
Sometimes	34 (15.3%)	2408 (18.2%)	167 (17.1%)		
Often	5 (2.3%)	269 (2.0%)	20 (2.0%)		
All of the time	0 (0%)	0 (0%)	0 (0%)		
**Sleep Chronotype**				0.672	0.828
Morning person	146 (65.8%)	8907 (67.3%)	654 (66.8%)		
Evening person	76 (34.2%)	4320 (32.7%)	325 (33.2%)		
**Sleep Duration**	7.2 (1.0)	7.1 (1.0)	7.1 (1.2)	0.331	0.347
**Sleeplesness**				**< 0.001**	**< 0.001**
Never/rarely	69 (31.1%)	2085 (15.8%)	145 (14.8%)		
Sometimes	92 (41.4%)	6403 (48.4%)	461 (47.1%)		
Usually	61 (27.5%)	4739 (35.8%)	373 (38.1%)		
**Snoring**				0.388	0.285
No	169 (76.1%)	9697 (73.3%)	708 (72.3%)		

Yes	53 (23.9%)	3530 (26.7%)	271 (27.7%)		
**Depression**				**0.0342**	0.742
None	199 (89.6%)	12210 (92.3%)	876 (89.5%)		
Single episode of probably major depression	5 (2.3%)	266 (2.0%)	22 (2.2%)		
Probable recurrent major depression (moderate)	9 (4.1%)	544 (4.1%)	52 (5.3%)		
Probable recurrent major depression (severe)	9 (4.1%)	207 (1.6%)	29 (3.0%)		
**Able to Confide**				0.759	0.291
Never or almost never	18 (8.1%)	1210 (9.1%)	116 (11.8%)		
Once every few months	18 (8.1%)	849 (6.4%)	51 (5.2%)		
About once a month	12 (5.4%)	884 (6.7%)	62 (6.3%)		
About once a week	26 (11.7%)	1810 (13.7%)	106 (10.8%)		
2-4 times a week	29 (13.1%)	1643 (12.4%)	106 (10.8%)		
Almost daily	119 (53.6%)	6831 (51.6%)	538 (55.0%)		
**Number in Household**	2.8 (1.3)	2.1 (1.0)	2.0 (1.1)	**< 0.001**	**< 0.001**
**Leisure/Social Activities**	1.1 (0.9)	1.3 (0.9)	1.2 (0.9)	**0.003**	**0.325**
**Frequency of Friend/Family Visits**				**0.007**	**0.027**
No friends/family outside household	1 (0.5%)	13 (0.1%)	2 (0.2%)		
Never or almost never	2 (0.9%)	98 (0.7%)	5 (0.5%)		
Once every few months	12 (5.4%)	624 (4.7%)	41 (4.2%)		
About once a month	36 (16.2%)	1418 (10.7%)	111 (11.3%)		
About once a week	77 (34.7%)	4122 (31.2%)	294 (30.0%)		
2-4 times a week	76 (34.2%)	4944 (37.4%)	373 (38.1%)		
Almost daily	18 (8.1%)	2008 (15.2%)	153 (15.6%)		
**Friendships Satisfaction**				0.207	0.537
Extremely unhappy	0 (0%)	5 (0.0%)	3 (0.3%)		
Very unhappy	0 (0%)	28 (0.2%)	2 (0.2%)		
Moderately unhappy	3 (1.4%)	259 (2.0%)	12 (1.2%)		
Moderately happy	71 (32.0%)	3461 (26.2%)	262 (26.8%)		
Very happy	107 (48.2%)	7399 (55.9%)	527 (53.8%)		
Extremely happy	41 (18.5%)	2075 (15.7%)	173 (17.7%)		
**Health Satisfaction**				**0.014**	**0.002**
Extremely unhappy	2 (0.9%)	53 (0.4%)	10 (1.0%)		
Very unhappy	3 (1.4%)	144 (1.1%)	16 (1.6%)		
Moderately unhappy	20 (9.0%)	809 (6.1%)	83 (8.5%)		
Moderately happy	87 (39.2%)	5776 (43.7%)	464 (47.4%)		
Very happy	84 (37.8%)	5555 (42.0%)	362 (37.0%)		
Extremely happy	26 (11.7%)	890 (6.7%)	44 (4.5%)		
**Work/Job Satisfaction**				< **.001**	**< 0.001**
Extremely unhappy	4 (1.8%)	595 (4.5%)	13 (1.3%)		
Very unhappy	15 (6.8%)	2703 (20.4%)	40 (4.1%)		
Moderately unhappy	15 (6.8%)	2736 (20.7%)	60 (6.1%)		
Moderately happy	79 (35.6%)	782 (5.9%)	178 (18.2%)		
Very happy	61 (27.5%)	526 (4.0%)	180 (18.4%)		
Extremely happy	24 (10.8%)	134 (1.0%)	47 (4.8%)		
I am not employed	24 (10.8%)	5751 (43.5%)	461 (47.1%)		

Table S4: Sample demographics of the female sample, separated by menopause status

	Premenopausal	Natural Menopause	Surgical Menopause	Age-Matched Males	p-value		
					**Pre vs Natural**	**Pre vs Surgical**	**Pre vs Males**
**N**	222	445	223	890			
**Age (years)**	53.4 (2.4)	53.8 (2.7)	54.1 (3.0)	53.8 (2.7)	0.071	**0.002**	**0.0485**
**Income**					0.304	0.190	**0.0129**
Less than 18,000	14 (6.3%)	31 (7.0%)	24 (10.8%)	46 (5.2%)			
18,000 to 30,999	28 (12.6%)	71 (16.0%)	37 (16.6%)	108 (12.1%)			
31,000 to 51,999	80 (36.0%)	126 (28.3%)	82 (36.8%)	226 (25.4%)			
52,000 to 100,000	72 (32.4%)	163 (36.6%)	58 (26.0%)	361 (40.6%)			
Greater than 100,000	28 (12.6%)	54 (12.1%)	22 (9.9%)	149 (16.7%)			
**Age completed full time education (years)**	19.3 (2.0)	19.1 (2.4)	18.7 (2.6)	19.2 (2.5)	0.354	**0.007**	0.618
**Diastolic Blood Pressure (mmHg)**	76.2 (8.3)	75.5 (9.0)	78.0 (8.4)	81.4 (9.5)	0.280	**0.026**	**< 0.001**
**Systolic Blood Pressure (mmHg)**	126.0 (14.7)	125.0 (14.2)	127.7 (15.4)	135.3 (14.0)	0.622	0.265	**< 0.001**
**Body Mass Index (BMI)**	26.2 (4.6)	25.7 (4.6)	27.0 (5.2)	27.2 (4.2)	0.123	0.059	**< 0.001**
**Frequency of walking**	5.6 (1.9)	5.6 (1.8)	5.6 (1.9)	5.5 (1.9)	0.682	0.787	0.457
**Frequency of moderate exercise**	3.6 (2.2)	3.8 (2.3)	3.5 (2.3)	3.6 (2.2)	0.327	0.730	0.984
**Frequency of vigorous exercise**	1.9 (1.9)	1.9 (1.8)	1.4 (1.6)	2.4 (1.9)	0.703	**0.016**	**< 0.001**
**Alcohol Intake Frequency**					0.576	**0.058**	**< 0.001**
Never	10 (4.5%)	26 (5.8%)	20 (9.0%)	59 (6.6%)			
Special occasions only	30 (13.5%)	63 (14.2%)	43 (19.3%)	55 (6.2%)			
One to three times a month	36 (16.2%)	64 (14.4%)	39 (17.5%)	101 (11.3%)			
Once or twice a week	72 (32.4%)	141 (31.7%)	63 (28.3%)	273 (30.7%)			
Three or four times a week	44 (19.8%)	107 (24.0%)	42 (18.8%)	266 (29.9%)			
Daily or almost daily	30 (13.5%)	44 (9.9%)	16 (7.2%)	136 (15.3%)			
**Ever Smoked**					0.170	0.316	**0.025**
No	126 (56.8%)	226 (50.8%)	115 (51.6%)	428 (48.1%)			
Yes	96 (43.2%)	219 (49.2%)	108 (48.4%)	462 (51.9%)			
**Past Tobacco Smoking**					0.142	0.432	**0.006**
I have never smoked	126 (56.8%)	226 (50.8%)	115 (51.6%)	425 (47.8%)			
Just tried once or twice	40 (18.0%)	68 (15.3%)	36 (16.1%)	137 (15.4%)			
Smoked occasionally	27 (12.2%)	68 (15.3%)	36 (16.1%)	124 (13.9%)			
Smoked on most or all days	29 (13.1%)	83 (18.7%)	36 (16.1%)	204 (22.9%)			
**Hearing Difficulty**					**0.009**	**0.012**	**0.005**
No	163 (73.4%)	280 (62.9%)	138 (61.9%)	561 (63.0%)			
Yes	59 (26.6%)	165 (37.1%)	85 (38.1%)	329 (37.0%)			
**Narcolepsy**					0.722	0.540	0.983
Never/rarely	183 (82.4%)	377 (84.7%)	189 (84.8%)	729 (81.9%)			
Sometimes	34 (15.3%)	58 (13.0%)	27 (12.1%)	140 (15.7%)			
Often	5 (2.3%)	10 (2.2%)	7 (3.1%)	21 (2.4%)			
All of the time	0 (0%)	0 (0%)	0 (0%)	0 (0%)			
**Sleep Chronotype**					0.902	0.296	0.255
Morning person	146 (65.8%)	289 (64.9%)	135 (60.5%)	546 (61.3%)			
Evening person	76 (34.2%)	156 (35.1%)	88 (39.5%)	344 (38.7%)			
**Sleep Duration**	7.2 (1.0)	7.0 (0.9)	6.9 (1.3)	7.0 (0.9)		**0.009**	**0.001**
**Sleeplessness**						**< 0.001**	0.676
Never/rarely	69 (31.1%)	76 (17.1%)	34 (15.2%)	290 (32.6%)			
Sometimes	92 (41.4%)	195 (43.8%)	92 (41.3%)	381 (42.8%)			
Usually	61 (27.5%)	174 (39.1%)	97 (43.5%)	219 (24.6%)			
**Snoring**					0.911	0.294	**<0.001**

No	169 (76.1%)	342 (76.9%)	159 (71.3%)	455 (51.1%)			
Yes	53 (23.9%)	103 (23.1%)	64 (28.7%)	435 (48.9%)			
**Depression**					0.507	0.302	**0.005**
None	199 (89.6%)	399 (89.7%)	187 (83.9%)	846 (95.1%)			
Single episode of probably major depression	5 (2.3%)	13 (2.9%)	6 (2.7%)	8 (0.9%)			
Probable recurrent major depression (moderate)	9 (4.1%)	23 (5.2%)	17 (7.6%)	26 (2.9%)			
Probable recurrent major depression (severe)	9 (4.1%)	10 (2.2%)	13 (5.8%)	10 (1.1%)			
**Able to Confide**					0.530	0.228	**0.026**
Never or almost never	18 (8.1%)	34 (7.6%)	22 (9.9%)	143 (16.1%)			
Once every few months	18 (8.1%)	21 (4.7%)	8 (3.6%)	55 (6.2%)			
About once a month	12 (5.4%)	24 (5.4%)	12 (5.4%)	51 (5.7%)			
About once a week	26 (11.7%)	60 (13.5%)	18 (8.1%)	81 (9.1%)			
2-4 times a week	29 (13.1%)	50 (11.2%)	27 (12.1%)	82 (9.2%)			
Almost daily	119 (53.6%)	256 (57.5%)	136 (61.0%)	478 (53.7%)			
**Number in Household**	2.8 (1.3)	2.7 (1.2)	2.5 (1.9)	2.9 (1.3)	0.127	**< 0.001**	0.915
**Leisure/Social Activities**	1.1 (0.9)	1.0 (0.8)	0.8 (0.8)	1.0 (0.8)	0.407	**0.004**	0.548
**Frequency of Friend/Family Visits**					0.636	0.772	**0.001**
No friends/family outside household	1 (0.5%)	0 (0%)	1 (0.4%)	2 (0.2%)			
Never or almost never	2 (0.9%)	6 (1.3%)	2 (0.9%)	18 (2.0%)			
Once every few months	12 (5.4%)	23 (5.2%)	18 (8.1%)	96 (10.8%)			
About once a month	36 (16.2%)	63 (14.2%)	35 (15.7%)	163 (18.3%)			
About once a week	77 (34.7%)	176 (39.6%)	68 (30.5%)	365 (41.0%)			
2-4 times a week	76 (34.2%)	137 (30.8%)	73 (32.7%)	198 (22.2%)			
Almost daily	18 (8.1%)	40 (9.0%)	26 (11.7%)	48 (5.4%)			
**Friendships Satisfaction**					0.759	0.820	**0.001**
Extremely unhappy	0 (0%)	0 (0%)	1 (0.4%)	3 (0.3%)			
Very unhappy	0 (0%)	1 (0.2%)	0 (0%)	10 (1.1%)			
Moderately unhappy	3 (1.4%)	11 (2.5%)	4 (1.8%)	51 (5.7%)			
Moderately happy	71 (32.0%)	142 (31.9%)	71 (31.8%)	314 (35.3%)			
Very happy	107 (48.2%)	219 (49.2%)	111 (49.8%)	419 (47.1%)			
Extremely happy	41 (18.5%)	72 (16.2%)	36 (16.1%)	93 (10.4%)			
**Health Satisfaction**					0.467	**0.003**	0.189
Extremely unhappy	2 (0.9%)	1 (0.2%)	4 (1.8%)	5 (0.6%)			
Very unhappy	3 (1.4%)	11 (2.5%)	7 (3.1%)	22 (2.5%)			
Moderately unhappy	20 (9.0%)	47 (10.6%)	25 (11.2%)	76 (8.5%)			
Moderately happy	87 (39.2%)	178 (40.0%)	100 (44.8%)	386 (43.4%)			
Very happy	84 (37.8%)	171 (38.4%)	82 (36.8%)	340 (38.2%)			
Extremely happy	26 (11.7%)	37 (8.3%)	5 (2.2%)	61 (6.9%)			
**Work/Job Satisfaction**					< **.001**	0.228	**< 0.001**
Extremely unhappy	4 (1.8%)	36 (8.1%)	5 (2.2%)	49 (5.5%)			
Very unhappy	15 (6.8%)	129 (29.0%)	15 (6.7%)	252 (28.3%)			
Moderately unhappy	15 (6.8%)	163 (36.6%)	31 (13.9%)	387 (43.5%)			
Moderately happy	79 (35.6%)	50 (11.2%)	71 (31.8%)	97 (10.9%)			
Very happy	61 (27.5%)	23 (5.2%)	57 (25.6%)	27 (3.0%)			
Extremely happy	24 (10.8%)	8 (1.8%)	16 (7.2%)	16 (1.8%)			
I am not employed	24 (10.8%)	36 (8.1%)	28 (12.6%)	62 (7.0%)			

Table S5: Sample demographics of the age-matched menopause sample, separated by sex and menopause status.

Analysis	LV	PLS-derived Score	Term	Estimate	95% CI	p value
**Whole Sample**	**LV1**	**Brain**	**Age**	**-0.2873**	**[-0.2970, -0.2775]**	**0.0000**
			**Sex**	**-0.2664**	**[-0.2800, -0.2527]**	**2.4593e-313**
			PRS	-0.0022	[-0.0124, 0.0080]	0.6683
			**Age x Sex**	**0.0246**	**[0.0110, 0.0381]**	**0.0004**
			Age x PRS	-0.0010	[-0.0108, 0.0089]	0.8502
			Sex x PRS	-0.0049	[-0.0185, 0.0088]	0.4855
			Age x Sex x PRS	-0.0117	[-0.0255, 0.0020]	0.0951
		**Behavior**	**Age**	**-2.1727**	**[-2.2445, -2.1010]**	**0.0000**
			**Sex**	**0.4697**	**[0.3690, 0.5703]**	**2.2051e-20**
			**PRS**	**-0.0855**	**[-0.1606, -0.0103]**	**0.0258**
			**Age x Sex**	**-0.6722**	**[-0.7724, -0.5720]**	**2.2281e-39**
			Age x PRS	-0.0222	[-0.0950, 0.0507]	0.5511
			Sex x PRS	0.0469	[-0.0539, 0.1478]	0.3616
			Age x Sex x PRS	-0.0025	[-0.1040, 0.0991]	0.9619
	**LV2**	**Brain**	**Age**	**0.0218**	**[0.0190, 0.0247]**	**8.0954e-50**
			**Sex**	**-0.2013**	**[-0.2053, -0.1972]**	**0.0000**
			**PRS**	**-0.0047**	**[-0.0077, -0.0017]**	**0.0022**
			**Age x Sex**	**0.0101**	**[0.0061, 0.0141]**	**8.4710e-7**
			**Age x PRS**	**-0.0030**	**[-0.0059, -0.0001]**	**0.0428**
			Sex x PRS	-0.0013	[-0.0053, 0.0028]	0.5314
			Age x Sex x PRS	-0.0014	[-0.0055, 0.0027]	0.4951
		**Behavior**	**Age**	**1.2003**	**[1.0977, 1.3028]**	**1.6791e-115**
			**Sex**	**-3.0287**	**[-3.1725, -2.8848]**	**0.0000**
			PRS	0.0482	[-0.0592, 0.1555]	0.3791
			**Age x Sex**	**0.8919**	**[0.7487, 1.0351]**	**3.3738e-34**
			Age x PRS	0.0303	[-0.0738, 0.1344]	0.5686
			Sex x PRS	-0.0496	[-0.1937, 0.0945]	0.5002
			Age x Sex x PRS	-0.0255	[-0.1706, 0.1197]	0.7309
**Male-Specific**	**LV1**	**Brain**	**Age**	**-0.2990**	**[-0.3092, -0.2889]**	**0.0000**
			PRS	-0.0021	[-0.0123, 0.0081]	0.6861
			Age x PRS	-0.0009	[-0.0111, 0.0094]	0.8676
		**Behavior**	**Age**	**-2.3373**	**[-2.4216, -2.2531]**	**0.0000**
			**PRS**	**-0.0875**	**[-0.1718, -0.0032]**	**0.0418**
			Age x PRS	-0.0189	[-0.1038, 0.0661]	0.6634
	**LV2**	**Brain**	Age	-0.0023	[-0.0060, 0.0015]	0.2435
			**PRS**	**0.0073**	**[0.0036, 0.0111]**	**0.0001**
			Age x PRS	0.0037	[-0.0001, 0.0075]	0.0584
		**Behavior**	**Age**	**0.2318**	**[0.0903, 0.3733]**	**0.0013**
			PRS	0.0372	[-0.1043, 0.1787]	0.6064
			Age x PRS	-0.0167	[-0.1593, 0.1260]	0.8189

**Female-Specific**	**LV1**	**Brain**	Age	0.0006	[-0.0108,0.0120]	0.9209
			Premenopause	-0.0297	[-0.3995,0.3400]	0.8748
			Surgical Menopause	0.0190	[-0.0239,0.0620]	0.3851
			PRS	-0.0072	[-0.0183,0.0040]	0.2099
			Age x Premenopause	-0.0213	[-0.2657,0.2230]	0.8640
			Age x Surgical Menopause	-0.0247	[-0.0669,0.0175]	0.2508
			Age x PRS	-0.0021	[-0.0135,0.0094]	0.7214
			PRS x Premenopause	-0.0492	[-0.4536,0.3553]	0.8116
			PRS x Surgical Menopause	-0.0012	[-0.0456,0.0433]	0.9589
			Age X PRS x Premenopause	-0.0536	[-0.3261,0.2190]	0.7001
			Age X PRS x Surgical Menopause	-0.0135	[-0.0592,0.0322]	0.5638
		**Behavior**	Age	-0.0228	[-0.1764,0.1308]	0.7709
			Premenopause	-2.8030	[-7.7914,2.1853]	0.2707
			**Surgical Menopause**	**-0.9328**	**[-1.5123,-0.3533]**	**0.0016**
			PRS	0.0325	[-0.1184,0.1833]	0.6730
			Age x Premenopause	-2.6378	[-5.9349,0.6592]	0.1168
			**Age x Surgical Menopause**	**-1.2827**	**[-1.8518,-0.7136]**	**1.003e-5**
			Age x PRS	-0.1168	[-0.2712,0.0376]	0.1380
			PRS x Premenopause	-0.3118	[-5.7681,5.1445]	0.9108
			PRS x Surgical Menopause	-0.1224	[-0.7223,0.4776]	0.6893
			Age X PRS x Premenopause	-0.1859	[-3.8628,3.4909]	0.9210
			Age X PRS x Surgical Menopause	0.2791	[-0.3376,0.8957]	0.3751
	**LV2**	**Brain**	Age	0.0010	[-0.0026,0.0046]	0.5962
			Premenopause	0.0124	[-0.1053,0.1301]	0.8362
			Surgical Menopause	-0.0006	[-0.0143,0.0131]	0.9292
			PRS	-0.0005	[-0.0041,0.0030]	0.7699
			Age x Premenopause	-0.0012	[-0.0790,0.0766]	0.9760
			Age x Surgical Menopause	-0.0053	[-0.0188,0.0081]	0.4373
			Age x PRS	-0.0002	[-0.0038,0.0035]	0.9174
			PRS x Premenopause	0.0418	[-0.0869,0.1706]	0.5242
			PRS x Surgical Menopause	-0.0051	[-0.0192,0.0091]	0.4830
			Age X PRS x Premenopause	0.0284	[-0.0584,0.1152]	0.5211
			Age X PRS x Surgical Menopause	-0.0026	[-0.0171,0.0120]	0.7303
		**Behavior**	Age	0.0637	[-0.0742,0.2015]	0.3653
			Premenopause	2.5027	[-1.9729,6.9784]	0.2731
			**Surgical Menopause**	**0.6473**	**[0.1273,1.1672]**	**0.0147**
			PRS	-0.0655	[-0.2008,0.0699]	0.3432
			Age x Premenopause	2.0171	[-0.9411,4.9752]	0.1814
			**Age x Surgical Menopause**	**0.4969**	**[-0.0137,1.0075]**	**0.0565**
			Age x PRS	-0.0112	[-0.1497,0.1273]	0.8742
			PRS x Premenopause	-2.7517	[-7.6472,2.1438]	0.2706

PRS x Surgical Menopause	-0.0628	[-0.6011,0.4755]	0.8192
Age X PRS x Premenopause	-2.2935	[-5.5924,1.0054]	0.1730
Age X PRS x Surgical Menopause	-0.3134	[-0.8667,0.2399]	0.2669

Table S6: Linear regression results predicting brain and behavioral scores from age, sex, and Alzheimer's disease polygenic risk score (PRS). Estimates represent standardized beta coefficients and 95% confidence intervals (CI). Models include interaction terms as indicated. Significant effects are highlighted by p-values ($p < 0.05$). Brain and behavior scores correspond to latent variables (LV1 and LV2) derived from partial least squares analysis.

S2. Supplementary Figures

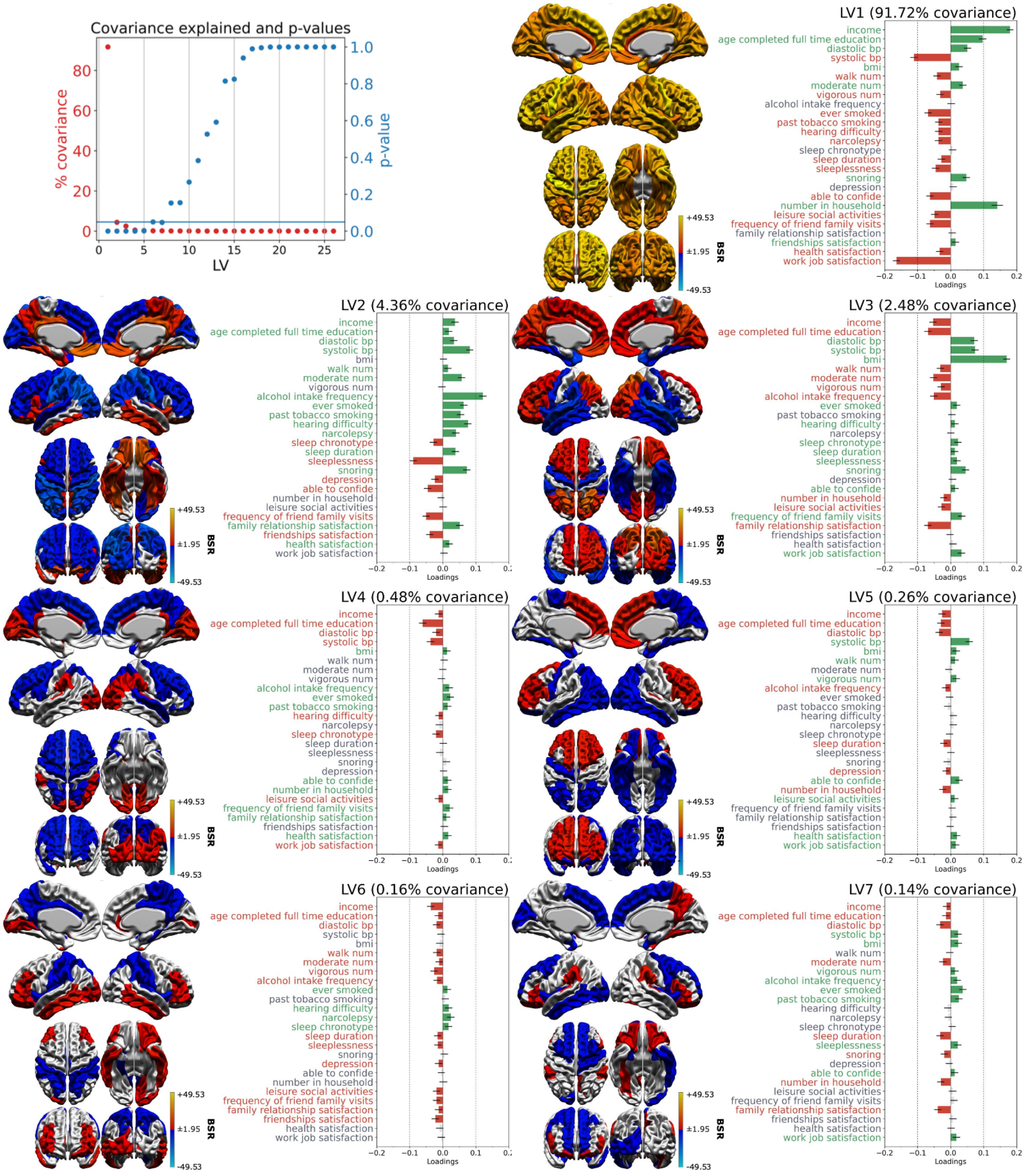

Figure S1: Latent Variables of Whole Sample Analysis. PLS found 7 significant ($p < 0.05$) latent variables (LVs) in the whole sample analysis. Covariance explained (red) and significance (blue) of each latent variable in the top left. The horizontal blue line represents the significance threshold of p-values at 0.05. The remaining plots showcase the morphometric (left) and behavioral (right) patterns for each LV. Warm colors on

morphometric maps indicate regions that vary positively with the LV, and cold colors indicate regions that vary negatively. Colored bars in behavioral loading plots indicate significant contributions to the clinical-behavioral signature, meaning maximal covariance with colored regions on the brain map. Green indicates a positive loading, and red indicates a negative loading. 95% confidence intervals are shown, and variables have non-significant contributions to the LV if their confidence intervals cross 0. (**BSR**: bootstrap ratio)

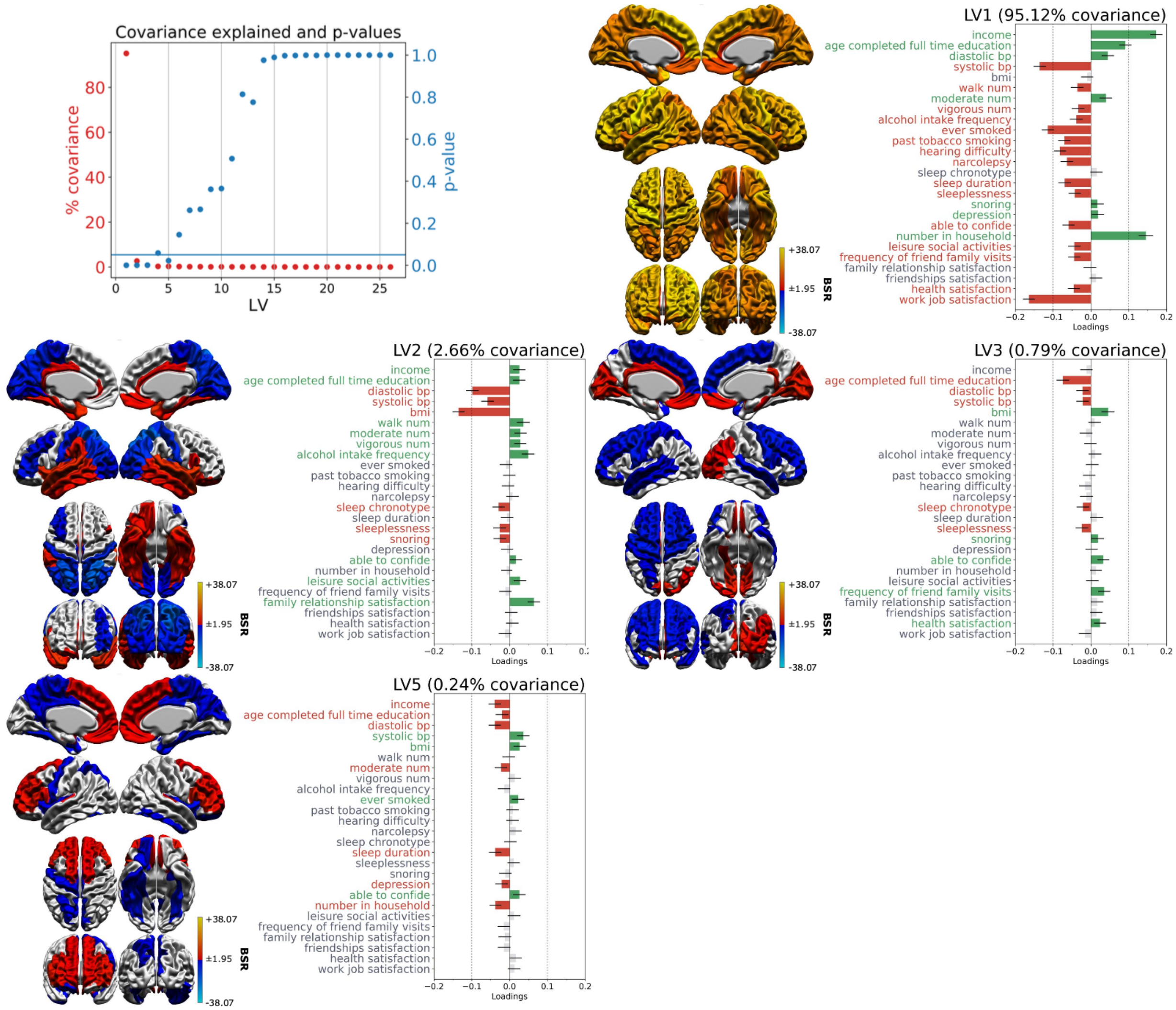

Figure S2: Latent Variables of male-specific analysis. PLS found 4 significant ($p < 0.05$) latent variables (LVs) in the male sample analysis. Covariance explained (red) and significance (blue) of each latent variable in the top left. The horizontal blue line represents the significance threshold of p-values at 0.05. The remaining plots showcase the morphometric (left) and behavioral (right) patterns for each LV. Warm colors on morphometric maps indicate regions that vary positively with the LV, and cold colors indicate regions that vary negatively. Colored bars in behavioral loading plots indicate significant contributions to the clinical-behavioral signature, meaning maximal covariance with colored regions on the brain map. Green indicates a positive loading, and red indicates a negative loading. 95% confidence intervals are shown, and variables have non-significant contributions to the LV if their confidence intervals cross 0. (**BSR**: bootstrap ratio)

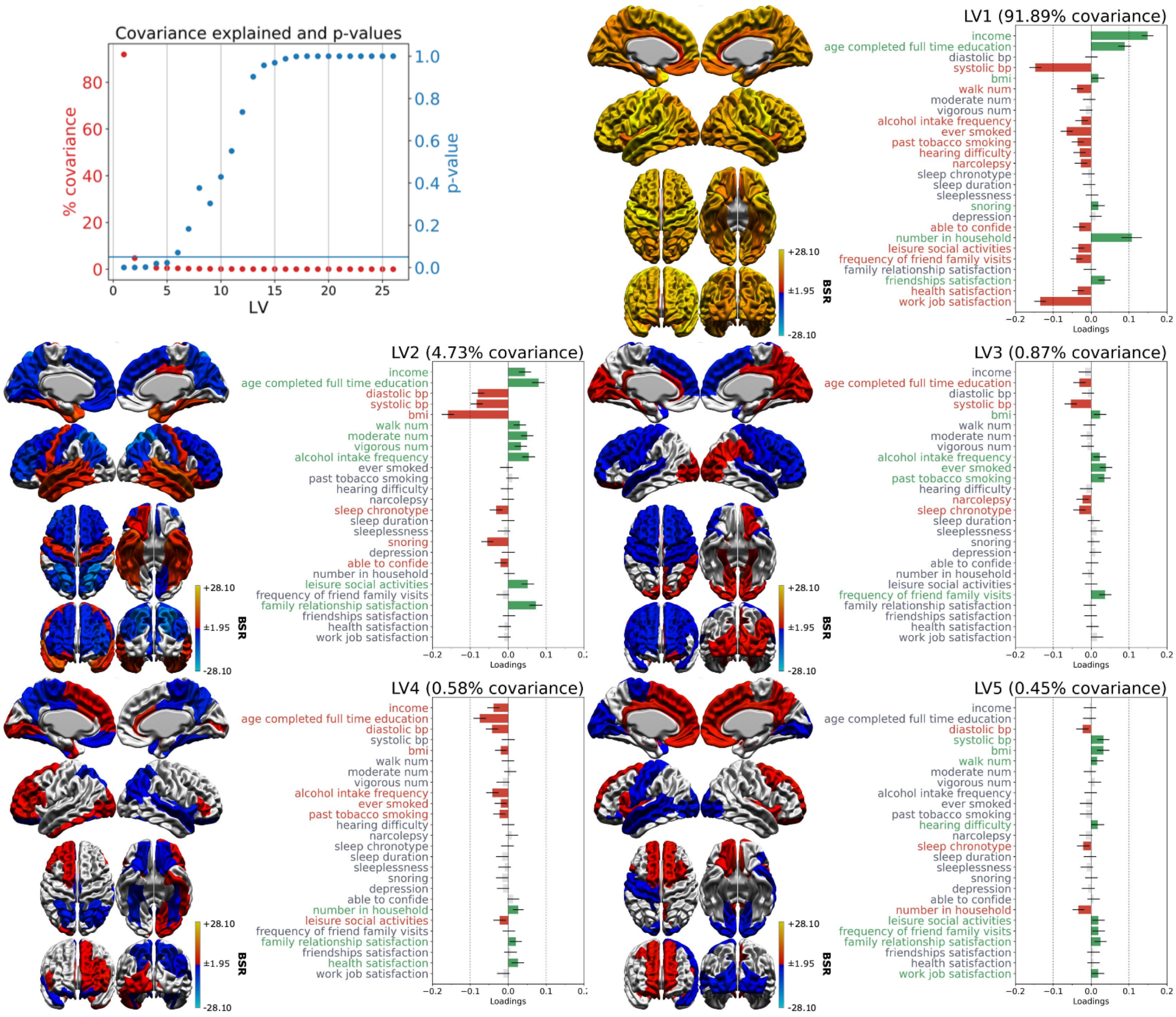

Figure S3: Latent Variables of female-specific analysis. PLS found 5 significant ($p < 0.05$) latent variables (LVs) in the female analysis. Covariance explained (red) and significance (blue) of each latent variable in the top left. The horizontal blue line represents the significance threshold of p-values at 0.05. The remaining plots showcase the morphometric (left) and behavioral (right) patterns for each LV. Warm colors on morphometric maps indicate regions that vary positively with the LV, and cold colors indicate regions that vary negatively. Colored bars in behavioral loading plots indicate significant contributions to the clinical-behavioral signature, meaning maximal covariance with colored regions on the brain map. Green indicates a positive loading, and red indicates a negative loading. 95% confidence intervals are shown, and variables have non-significant contributions to the LV if their confidence intervals cross 0. (**BSR**: bootstrap ratio)

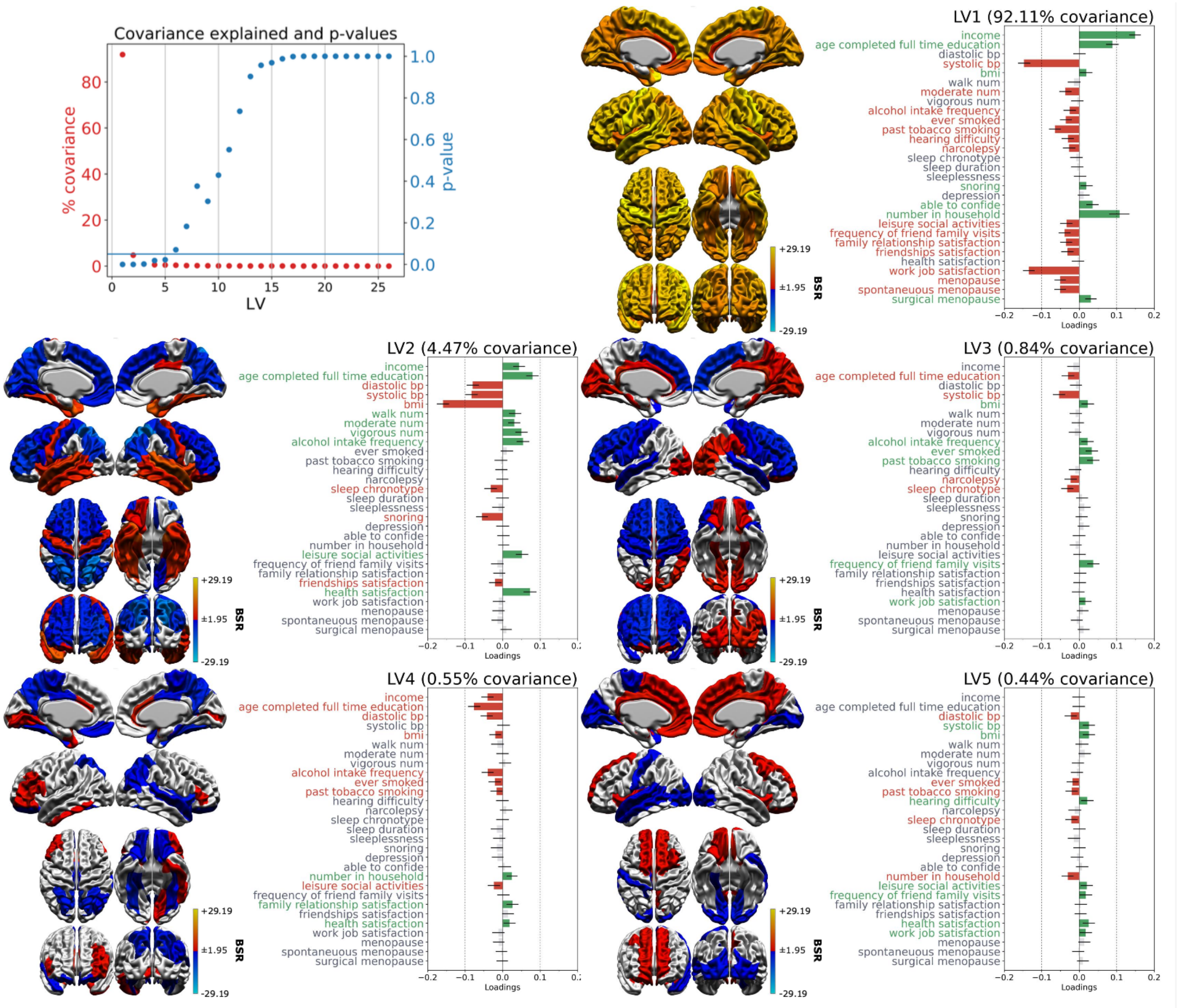

Figure S4: Latent Variables of female-specific analysis with inclusion of menopause variables. PLS found 5 significant ($p < 0.05$) latent variables (LVs) in the female menopause-related analysis. Covariance explained (red) and significance (blue) of each latent variable in the top left. The horizontal blue line represents the significance threshold of p-values at 0.05. The remaining plots showcase the morphometric (left) and behavioral (right) patterns for each LV. Warm colors on morphometric maps indicate regions that vary positively with the LV, and cold colors indicate regions that vary negatively. Colored bars in behavioral loading plots indicate significant contributions to the clinical-behavioral signature, meaning maximal covariance with colored regions on the brain map. Green indicates a positive loading, and red indicates a negative loading. 95% confidence intervals are shown, and variables have non-significant contributions to the LV if their confidence intervals cross 0. (**BSR**: bootstrap ratio)

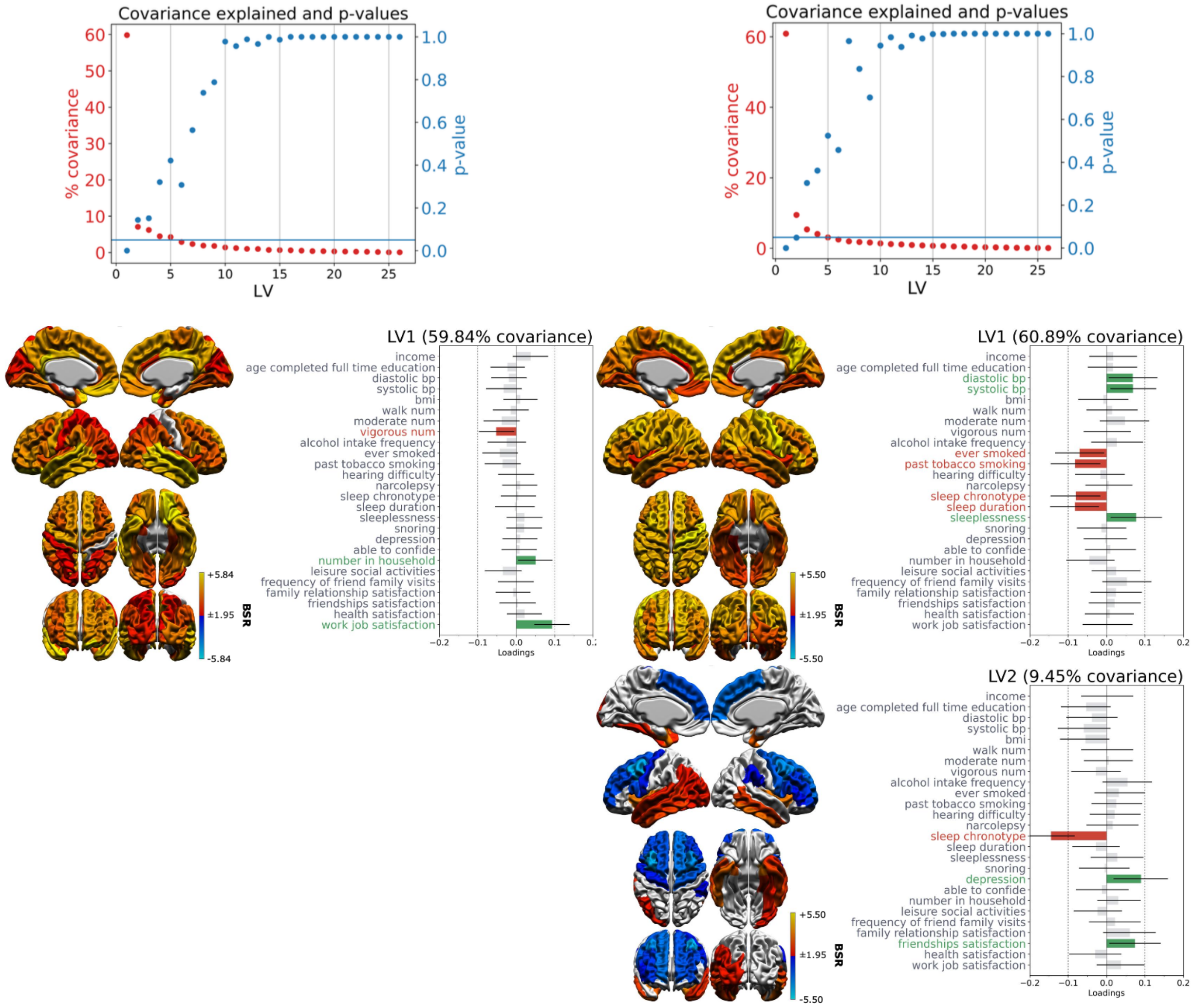

Figure S5: Significant latent variables of menopause-matched and age-matched female groups and repeated analysis with the inclusion of the age-matched male group. Using the age-matched and menopause-status-matched subsample, PLS found 1 significant ($p < 0.05$) latent variable (LV) in the analysis with only the females (left), and two significant LVs with the inclusion of the age-matched male group (right). Covariance explained (red) and significance (blue) of each latent variable in the top left. The horizontal blue line represents the significance threshold of p-values at 0.05. The remaining plots showcase the morphometric (left) and behavioral (right) patterns for each LV. Warm colors on morphometric maps indicate regions that vary positively with the LV, and cold colors indicate regions that vary negatively. Colored bars in behavioral loading plots indicate significant contributions to the clinical-behavioral signature, meaning maximal covariance with colored regions on the brain map. Green indicates a positive loading, and red indicates a negative loading. 95% confidence intervals are shown, and variables have non-significant contributions to the LV if their confidence intervals cross 0. (**BSR**: bootstrap ratio)

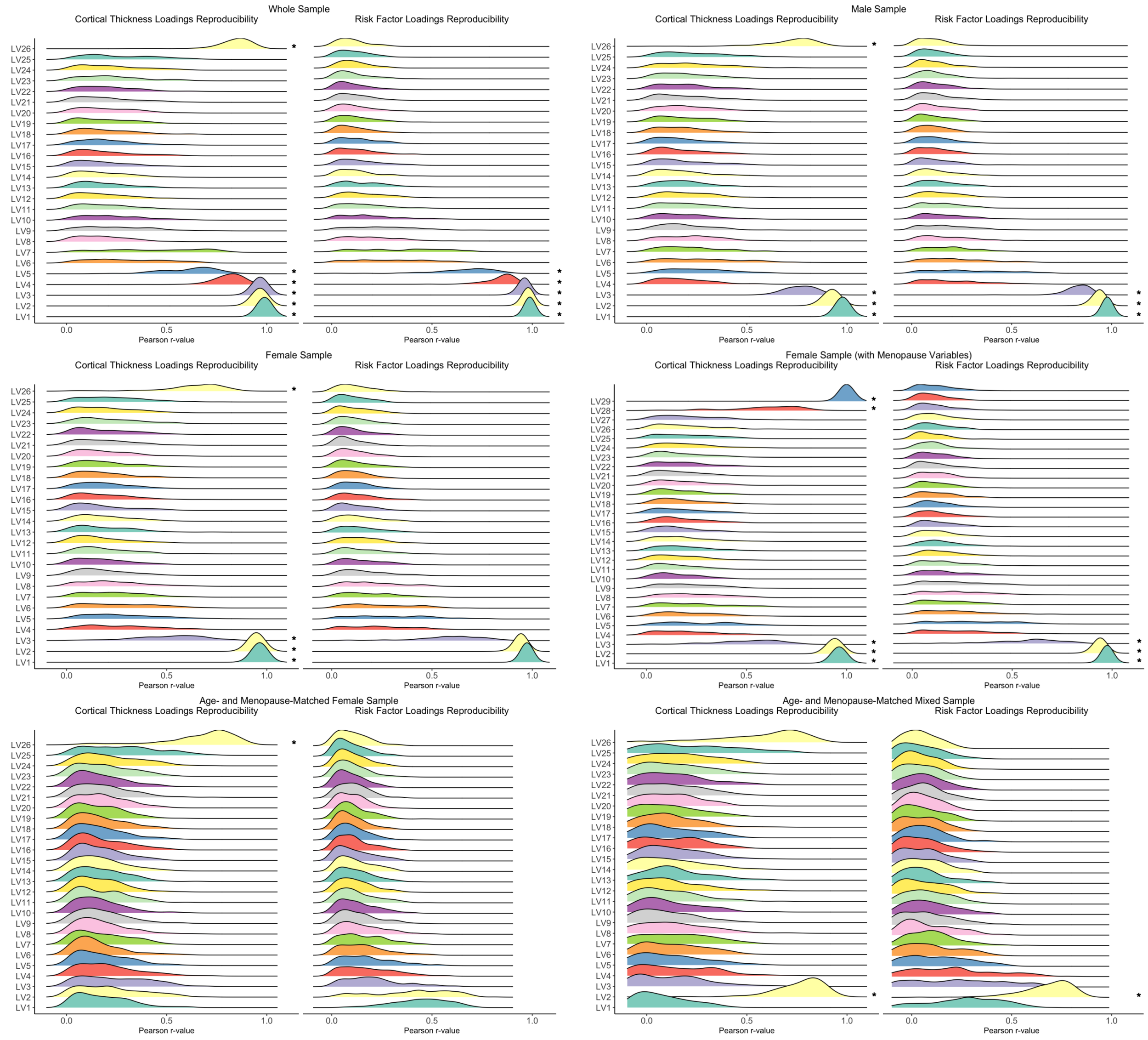

Figure S6: Distributions from split-half stability analysis for each PLS model. Split-half resampling analysis found varying levels of stability within each PLS model. In each plot, each row represents the distribution of Pearson correlation coefficients between respective loadings from each split-half analysis. Distributions centered around zero indicate minimal correspondence between loadings from each split-half analysis, indicating higher sensitivity of participant characteristics. Asterisks indicate the LVs that showed a significant distribution (Z-score > 1.96).

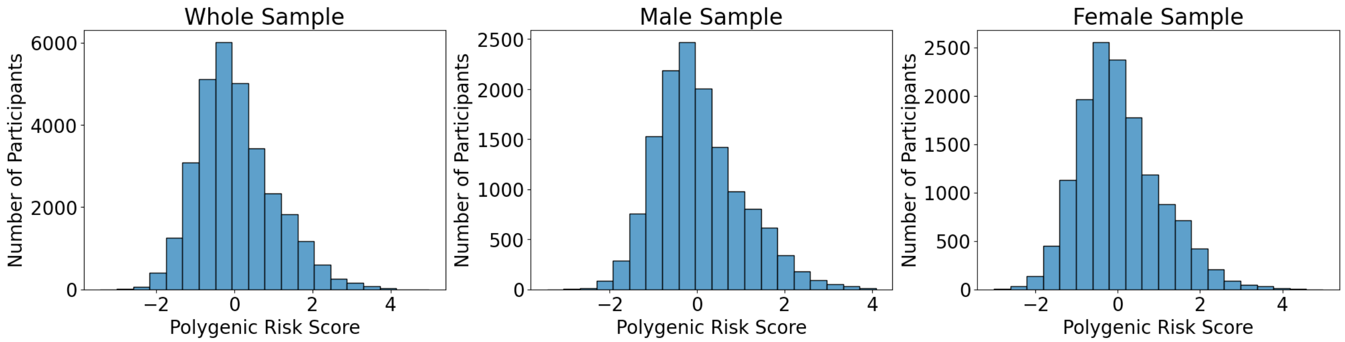

Figure S7: Distribution of PRS Scores of participants in main analyses. Distribution of PRS for Alzheimer's Disease of participants in the main analyses.

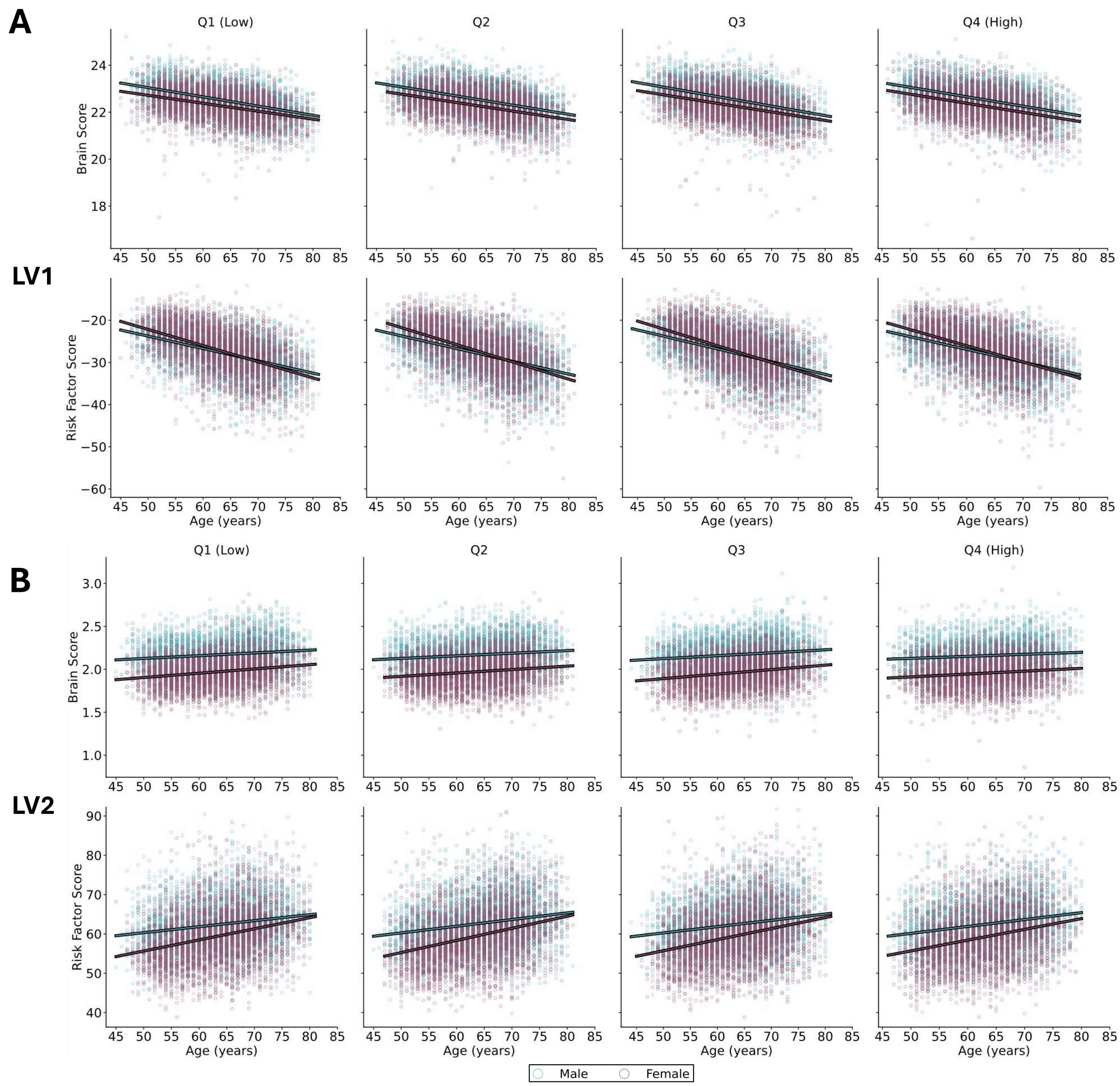

Figure S8: Age-related trajectories of brain and behavioral scores stratified by polygenic risk score (PRS) quartile. Linear regression models were fit separately within each PRS quartile (Q1–Q4) and sex to estimate the relationship between age and brain/behavioral scores. Each facet displays the regression lines and 95% confidence interval within one PRS quartile, allowing visual comparison of age-related trends across the PRS distribution. Results are shown separately for brain scores and behavioral risk factor scores, highlighting how polygenic risk may influence age-related changes in brain-behavior phenotypes.

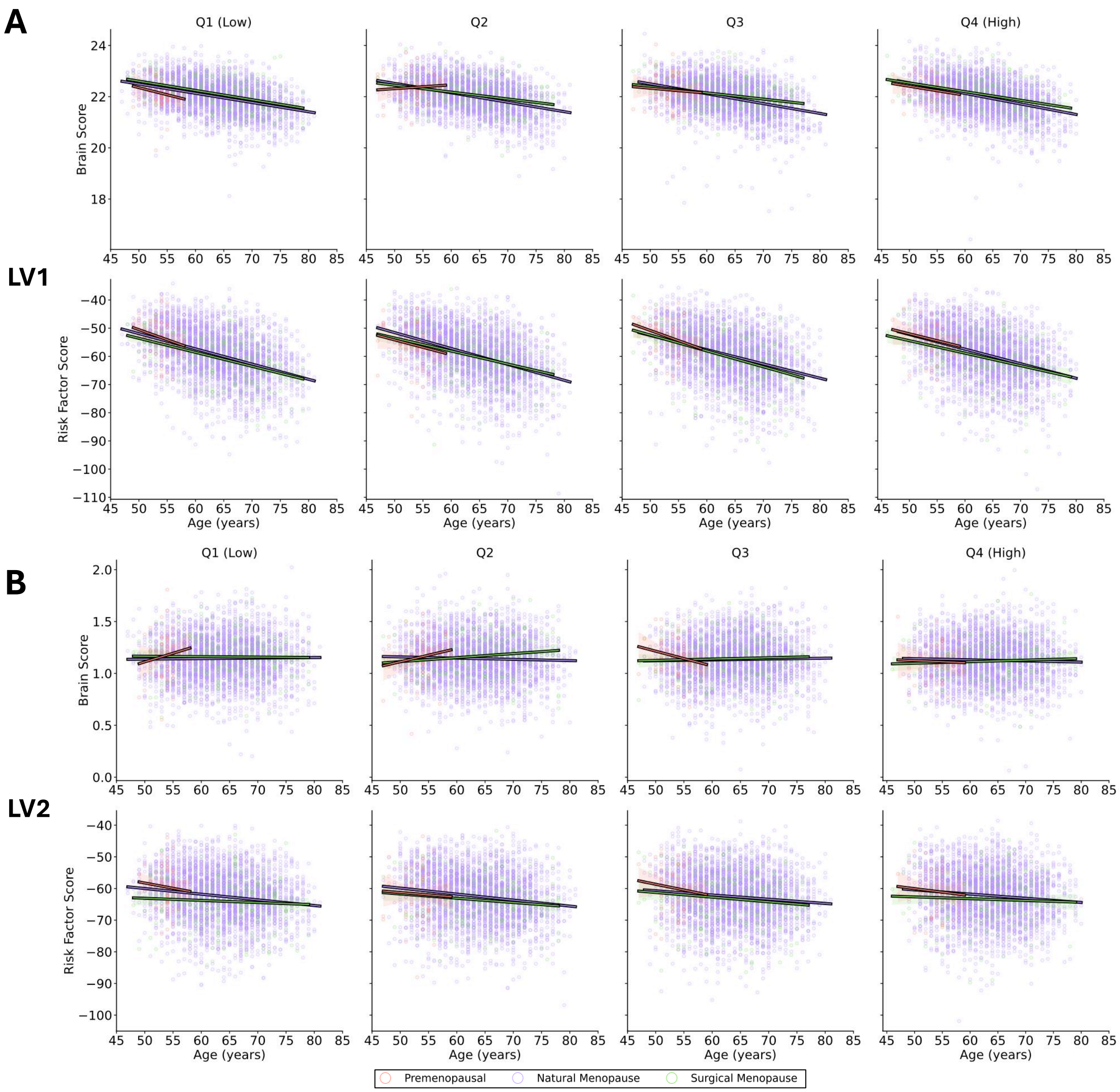

Figure S9: Age-related trajectories of brain and behavioral scores stratified by polygenic risk score (PRS) quartile. Linear regression models were fit separately within each PRS quartile (Q1–Q4) and menopausal group to estimate the relationship between age and brain/behavioral scores. Each facet displays the regression lines and 95% confidence interval within one PRS quartile, allowing visual comparison of age-related trends across the PRS distribution. Results are shown separately for brain scores and behavioral risk factor scores.

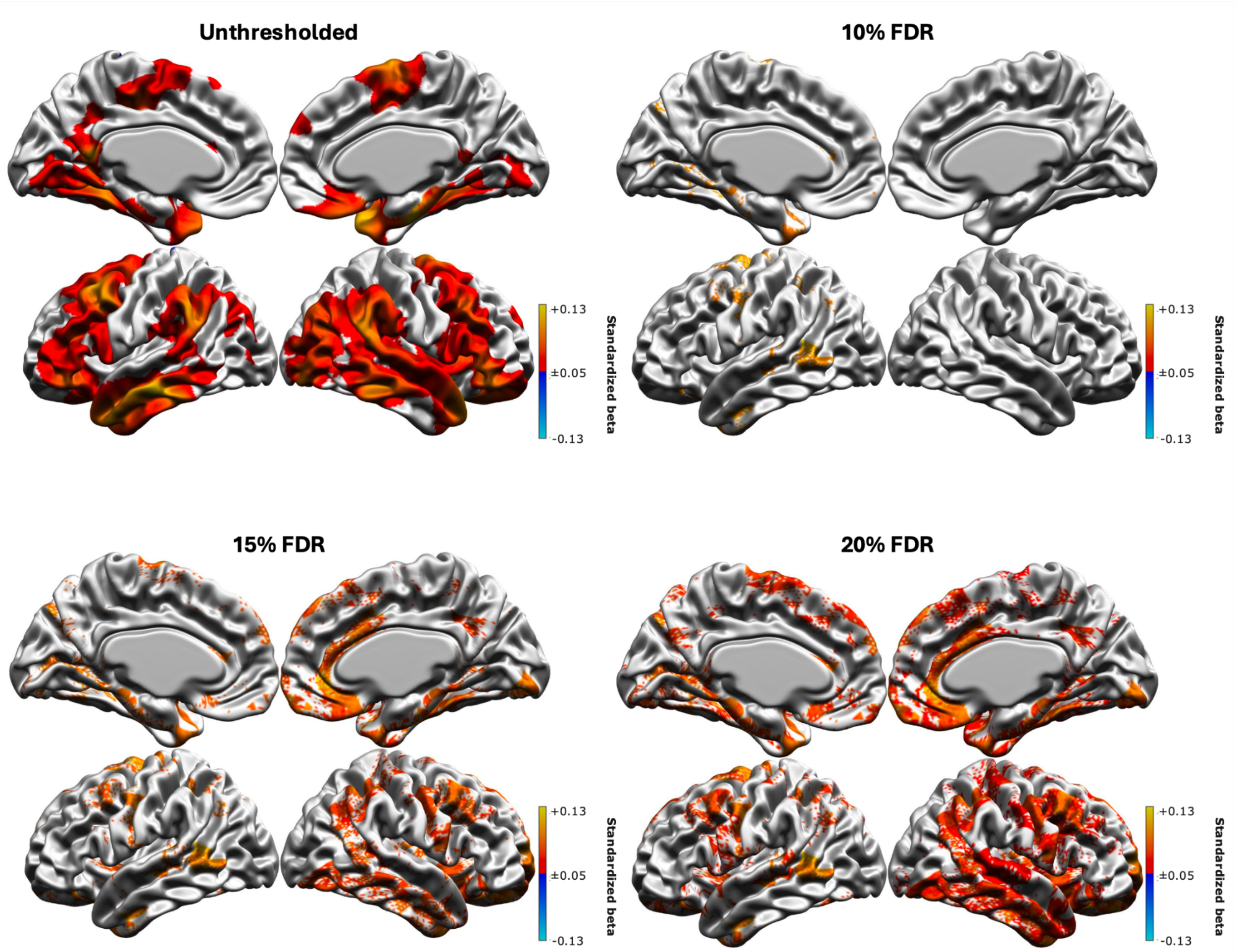

Figure S10. Surface maps showing standardized beta coefficients for the interaction between age and menopause status on cortical thickness.

Top left: Unthresholded map illustrating the full spatial distribution of effects.

Top right, bottom left, and bottom right: Maps thresholded at FDR-corrected q-values of 10%, 15%, and 20%, respectively.

These results demonstrate the spatial extent and consistency of the observed associations across multiple statistical thresholds, supporting the robustness of the findings while acknowledging the increased variability and reduced power in the menopause-stratified sample.
